# Functional Analysis of Metabolic Channeling and Regulation in Lignin Biosynthesis: A Computational Approach

**DOI:** 10.1371/journal.pcbi.1002769

**Published:** 2012-11-08

**Authors:** Yun Lee, Luis Escamilla-Treviño, Richard A. Dixon, Eberhard O. Voit

**Affiliations:** 1 The Interdisciplinary Bioengineering Program, Georgia Institute of Technology and Emory University, Atlanta, Georgia, United States of America; 2 BioEnergy Sciences Center (BESC), Oak Ridge, Tennessee, United States of America; 3 Plant Biology Division, Samuel Roberts Noble Foundation, Ardmore, Oklahoma, United States of America; 4 The Wallace H. Coulter Department of Biomedical Engineering, Georgia Institute of Technology and Emory University, Atlanta, Georgia, United States of America; The Pennsylvania State University, United States of America

## Abstract

Lignin is a polymer in secondary cell walls of plants that is known to have negative impacts on forage digestibility, pulping efficiency, and sugar release from cellulosic biomass. While targeted modifications of different lignin biosynthetic enzymes have permitted the generation of transgenic plants with desirable traits, such as improved digestibility or reduced recalcitrance to saccharification, some of the engineered plants exhibit monomer compositions that are clearly at odds with the expected outcomes when the biosynthetic pathway is perturbed. In *Medicago*, such discrepancies were partly reconciled by the recent finding that certain biosynthetic enzymes may be spatially organized into two independent channels for the synthesis of guaiacyl (G) and syringyl (S) lignin monomers. Nevertheless, the mechanistic details, as well as the biological function of these interactions, remain unclear. To decipher the working principles of this and similar control mechanisms, we propose and employ here a novel computational approach that permits an expedient and exhaustive assessment of hundreds of minimal designs that could arise *in vivo*. Interestingly, this comparative analysis not only helps distinguish two most parsimonious mechanisms of crosstalk between the two channels by formulating a targeted and readily testable hypothesis, but also suggests that the G lignin-specific channel is more important for proper functioning than the S lignin-specific channel. While the proposed strategy of analysis in this article is tightly focused on lignin synthesis, it is likely to be of similar utility in extracting unbiased information in a variety of situations, where the spatial organization of molecular components is critical for coordinating the flow of cellular information, and where initially various control designs seem equally valid.

## Author Summary

The organization of cooperating enzymes into complexes is a pervasive feature of metabolism. In particular, this phenomenon has been shown to participate in the regulation of flux through the networks of both primary and secondary metabolism in plants. It remains a challenging task to unravel the organizing principles of such “metabolic channels,” which can be temporary or persistent, and to understand their biological function. In this article, we analyze metabolic channels in the biosynthetic pathway of lignin, a complex polymer that stiffens and fortifies secondary cell walls within woody tissues. This system is well suited because the present analysis can be based on a computational, experimentally validated model demonstrating that several enzymes are spatially associated into channels specific for the production of two lignin monomers. To characterize the functioning of these channels, we develop a novel computational approach that is capable of identifying interesting structural and regulatory features of metabolic channeling and permits the formulation of targeted and readily testable hypotheses. Since the spontaneous or controlled assembly of molecules into functional units is known to occur in many biological contexts where information flow is tightly coordinated, the proposed approach might have broad applications in the field of computational systems biology.

## Introduction

Lignin is a phenolic heteropolymer in the secondary cell walls of vascular plants. It is derived mainly from three hydroxycinnamyl alcohol monomers, namely *p-*coumaryl, coniferyl, and sinapyl alcohols, which, when incorporated into the lignin polymer, give rise to *p-*hydroxyphenyl (H), guaiacyl (G), and syringyl (S) subunits, respectively. The development of lignin biosynthesis is considered to be one of the key factors that allowed vascular plants to dominate the terrestrial ecosystem [1]. This evolutionary advantage is in part due to the fact that lignin, when deposited in the cell wall, contributes to the structural integrity of the cell, facilitates transport of water and minerals through the tracheary elements, and serves as a defensive barrier against pathogens and herbivores [2].

In addition to lignin, the secondary cell walls of vascular plants contain several polysaccharides, such as cellulose and hemicellulose. Extraction of these polymers from lignocellulosic biomass for biofuel production has attracted extensive interest partly because exploitation of this source of fermentable sugars could minimize the competition for food, which has been criticized in the case of corn- or sugarcane-based biofuel production. However, the natural resistance of lignocellulosic biomass to enzymatic or microbial deconstruction has rendered the task of generating sustainable and cost effective biofuels from lignocellulosic feedstocks very challenging. While impressive advances have been made toward the reduction of biomass recalcitrance [3], [4], it was also shown that the amount of fermentable sugars released through chemical and enzymatic treatments is inversely proportional to that of lignin present in biomass, and that some transgenic plants with reduced lignin content yield up to twice as much sugar from their stems as wild-type plants [5]. These observations suggest that lignin biosynthesis may be targeted for generating engineered crops with reduced recalcitrance. The challenge of this task derives from the fact that a rational design of less recalcitrant varieties would require a thorough, multi-level understanding of the lignin biosynthesis in wild- type plants, which we do not yet have. Such an understanding would include a grasp of the system of interactions between the enzyme-encoding genes, proteins and metabolites involved in the biosynthesis of lignin, as well as details of the regulation of this multi-tiered system.

**Figure 1:**
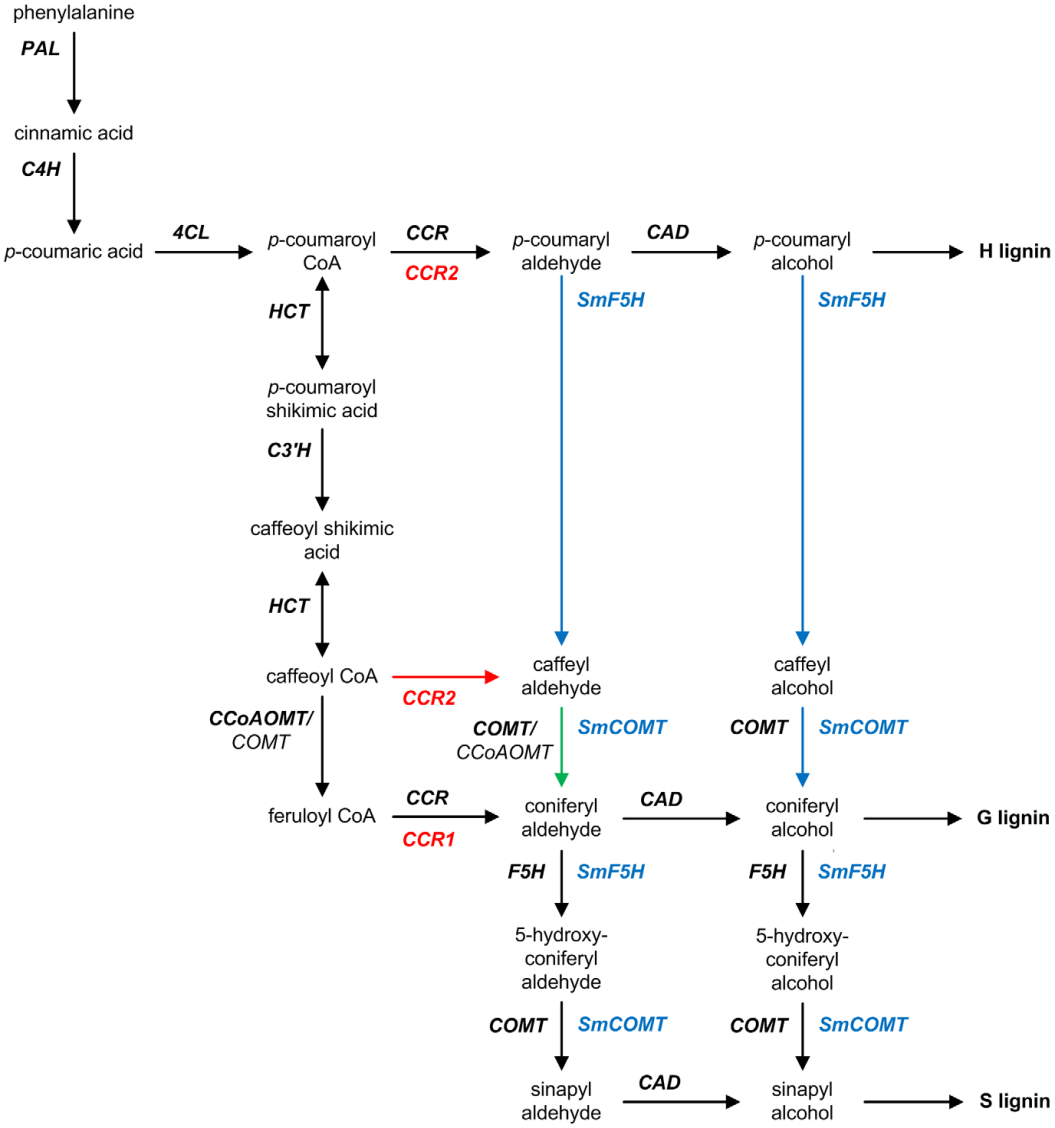
Generic pathway diagram of lignin biosynthesis with species-specific extensions. The widely accepted generic pathway consists of black arrows. It leads to the biosynthesis of three hydroxycinnamyl alcohol monomers that in turn give rise to *p*-hydroxyphenyl (H), guaiacyl (G), and syringyl (S) subunits of lignin. Listed next to each reaction arrow are the catalyzing enzymes, which are highlighted in bold if considered major. The lycophyte *Selaginella moellendorffi* contains two bi-functional enzymes, *Sm*F5H and *Sm*COMT, which are shown in blue. Co-expression of these two enzymes would permit *S. moellendorffi* to synthesize coniferyl and sinapyl alcohol directly from *p*-coumaryl aldehyde and *p*-coumaryl alcohol. By contrast, *Medicago truncatula* has two functionally distinct isoforms of CCR, which are shown in red. The green arrow that connects caffeyl aldehyde to coniferyl aldehyde denotes the only non-canonical reaction that is likely to be functional in both *S. moellendorffi* and *M. truncatula*. Abbreviations: CAD, cinnamyl alcohol dehydrogenase; CCoAOMT, caffeoyl CoA *O*-methyltransferase; CCR, cinnamoyl CoA reductase; C3′H, *p*-coumaroyl shikimate 3′-hydroxylase; C4H, cinnamate 4-hydroxylase; 4CL, 4-coumarate:CoA ligase; COMT, caffeic acid *O*-methyltransferase; F5H, ferulate 5-hydroxylase; HCT, hydroxycinnamoyl-CoA:shikimate hydroxycinnamoyl transferase; PAL, L-phenylalanine ammonia-lyase.

The metabolic scaffold for the biosynthesis of the three building blocks of lignin originally was seen as a grid-like structure [6], but this initial structure has been revised and refined and is now understood as an essentially linear pathway with only a few branch points (Figure 1). Although this generic pathway structure is now widely accepted, it has become clear that different lineages of vascular plants have evolved variants that engage distinct biosynthetic strategies. An interesting example is the model legume *Medicago truncatula*, where the characterization of two distinct cinnamoyl CoA reductases, CCR1 and CCR2, has suggested parallel routes from caffeoyl CoA to coniferyl aldehyde (Figure 1) [7]. A more unusual case is the lycophyte *Selaginella moellendorffi*. Functional analyses of the two enzymes recently discovered from this species, *Sm*F5H and *Sm*COMT, support the notion that *S. moellendorffi* may have adopted a non-canonical pathway from that in angiosperms to synthesize coniferyl and sinapyl alcohol (Figure 1) [8], [9], [10].

Given such variations, it would appear reasonable to consider genus- or species-specific similarities and differences. However, such data are seldom available, and even if a customized pathway structure can be established, its regulation often remains obscure. This shortcoming tends to become evident with new, precise data. For instance, experiments using genetically modified *M. truncatula* lines with reduced CCR1 activity exhibited an unexplainable decrease in the ratio of S to G lignin over wild type [7]. Such discrepancies between expectation and observation suggest that the currently accepted pathway diagrams may require further revisions that include regulatory mechanisms affecting the physiological outcome when the pathway is perturbed.

The focus of this article is an assessment of such a regulatory system associated with lignin biosynthesis in *Medicago*. This genus includes model species like *M. truncatula*, as well as alfalfa (*Medicago sativa* L.), an important forage legume. *Medicago* is particularly suited for these studies, because comparatively extensive information is available. For instance, a detailed dataset was established that characterized different lines in which seven lignin biosynthetic enzymes were independently down-regulated, and the resulting lignin content and monomer composition were determined in several stem segments [11]. In a recent study, we demonstrated that these types of data contain substantial, although hidden, information. In particular, we used these data to show that certain enzymes may co-localize and/or assemble into two independent channels for the synthesis of G and S lignin, and that salicylic acid acts as a potential regulatory molecule for the lignin biosynthetic pathway [12].

Although these earlier results provided significant insights into the mechanisms of regulation in this pathway, several critical questions, especially regarding the biological function as well as the operating mode of the channels, remain unanswered: For instance, are these channels always active *in vivo*? Are they sufficient to explain all available data in *Medicago*? Is there crosstalk between them, and if so, how is it organized? Exploring all pertinent scenarios associated with such questions would be experimentally intractable because they are simply too numerous.

**Figure 2:**
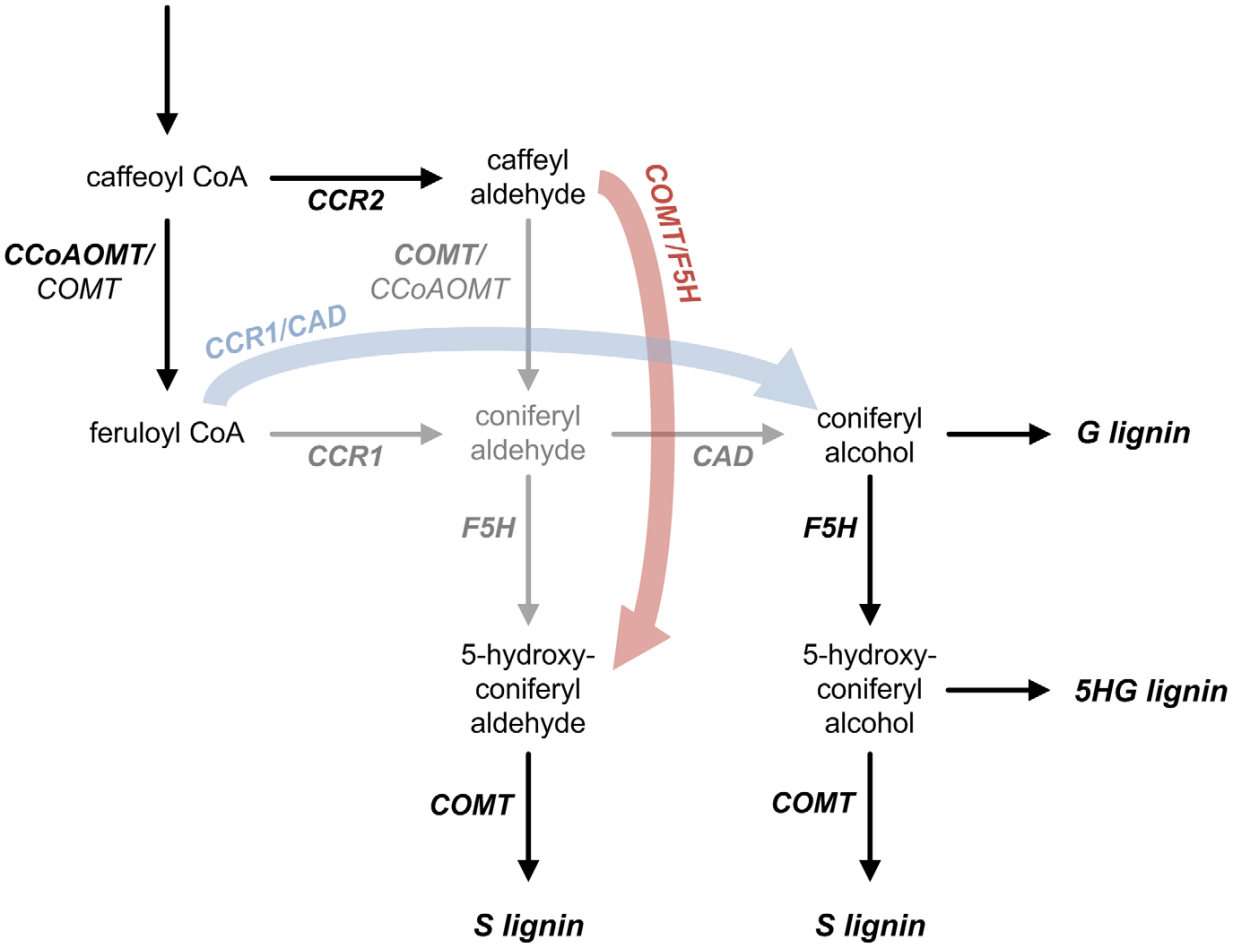
Scaffold of topological configurations. The relevant metabolites and enzymatic reactions (arrows) for the biosynthesis of guaiacyl (G), 5-hydroxyguaiacyl (5HG), and syringyl (S) lignin monomers are shown in black, if they are included in all topological configurations, or gray, if they are included in only some specific configurations. G and S channels are represented as blue and red arrows, respectively. Notice that 5-hydroxyconiferyl alcohol is allowed to be incorporated into lignin polymer as 5HG subunit because this actually occurs when COMT is down-regulated [41]. Enzymes are highlighted in bold and italics.

**Figure 3:**
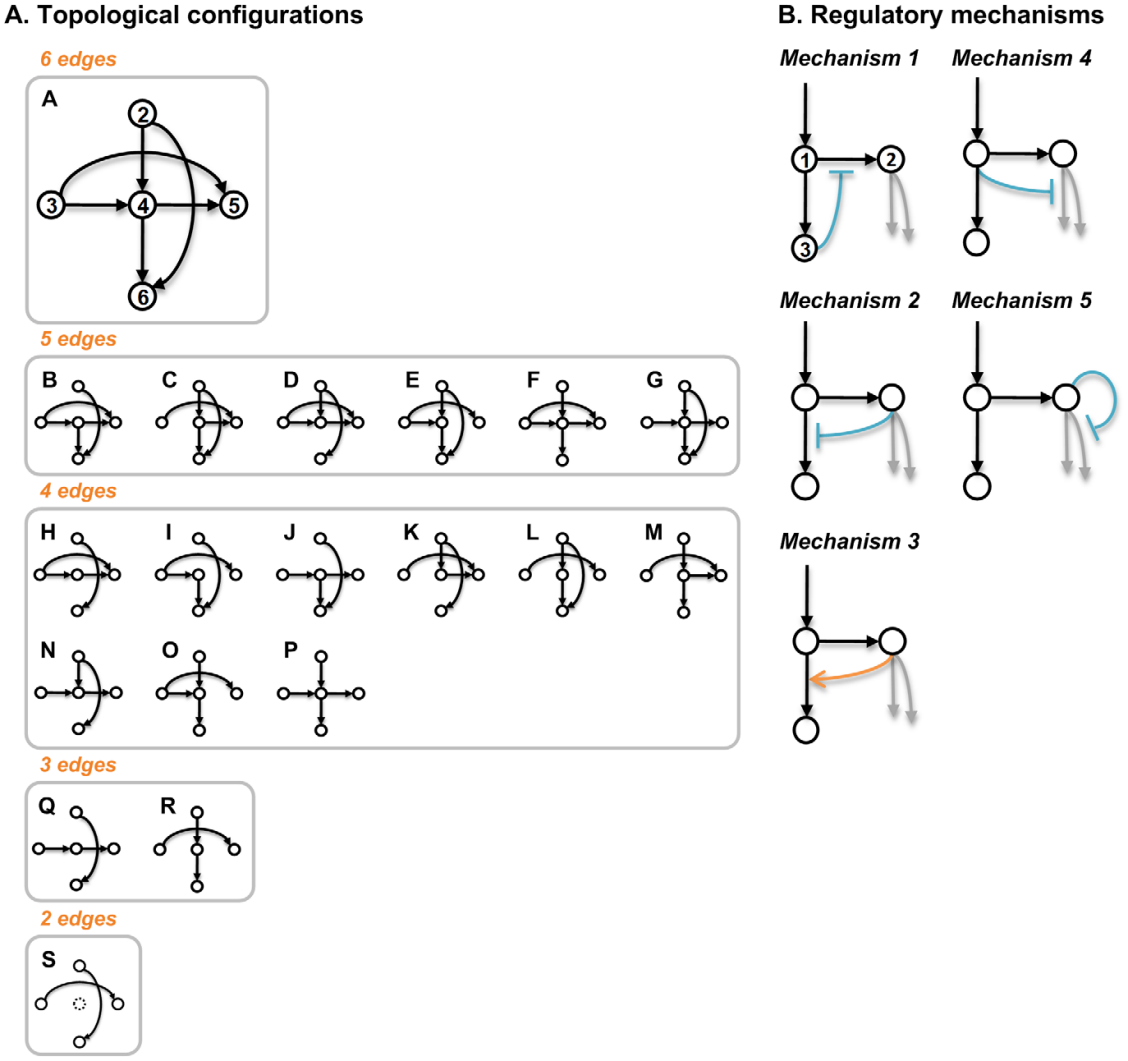
Lists of topological configurations and regulatory mechanisms. Panel A: The topological configurations differ in their numbers of edges. Panel B: The orange arrows represent activation processes, whereas the blocked lines (aqua) represent inhibition processes. Arrows colored in gray are reactions included in only some specific topological configurations. Metabolite names: 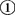
1 caffeoyl CoA; 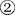, caffeyl aldehyde; 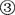 feruloyl CoA; 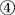 coniferyl aldehyde; 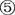 coniferyl alcohol; 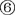 5-hydroxyconiferyl aldehyde.

Instead, we present here a novel computational approach to investigate exhaustively all regulatory schemes involving the key reactions associated with G and S channels in the lignin biosynthetic pathway (Figure 2). The specific hypothesis is that the formerly postulated and validated channels may have two different modes of operation. Either they are *permanent* in a sense that the component enzymes are persistently assembled into a complex; such a complex could be realized through membrane co-localization, thereby ensuring that the corresponding alcohol is always synthesized. As an alternative, the channels could be *facultative*, thereby displaying a functionality that depends on the sub-cellular localization of the component enzymes and the metabolic milieu. This hypothesis, in turn, leads to 19 possible topological configurations (Figure 3A). For each of these topologies, we consider an additional level of regulation, involving individual or combined regulatory mechanisms that may serve as a means of “crosstalk” between the two channels (Figure 3B). The emphasis of this approach is on mechanisms at the metabolic level, but one must not forget that the transcriptional network governing the system could be involved in regulation of the pathway as well [13].

The goal is thus to assess and compare the functionality of all given combinations of topological configurations and crosstalk patterns, each of which we call a *design*. To obtain insights that are independent of parameter choices, we constructed for each design a library of 100,000 loosely constrained dynamic models and tested each of them against the observed ratios of S to G lignin in four lignin-modified *Medicago* lines. The resulting analysis of hundreds of designs and millions of models led to the intriguing hypothesis that either a single activation mechanism or a dual-inhibition mechanism lies at the core of all experimentally supported designs. The former mechanism was not supported by an *in vitro* enzyme assay, while the latter is consistent with several lines of evidence from *Medicago* and other species. As an added insight, the analysis suggested that functionality of the G lignin channel is more important than that of the S lignin channel. Overall, these findings not only enrich our current understanding of how lignin biosynthesis is regulated, but they also demonstrate the possible application of the proposed approach in entirely different biological scenarios where the task is to identify true regulatory circuit among many theoretically feasible designs that depend on the functionality and localization of interacting molecules.

## Results

### Enumeration of circuit designs

The base scaffold on which the different topological variants were built is shown in Figure 2. It consists of all relevant steps in the lignin biosynthetic pathway that possibly affect the relative amounts of G and S lignin. Prior work [12] provided evidence that CCR1 and cinnamyl alcohol dehydrogenase (CAD) may organize into a functional complex through which the substrate coniferyl aldehyde is transferred from CCR1 to CAD without much leakage, thereby acting as a channel leading specifically to the synthesis of G lignin. Similarly, caffeic acid *O*-methyltransferase (COMT) and ferulate 5-hydroxylase (F5H) were suggested to form an analogous complex contributing specifically to the synthesis of S lignin. These two complexes, which we called G and S lignin channels, are represented in Figure 2 as two directed edges, one linking feruloyl CoA and coniferyl alcohol (G channel) and the other linking caffeyl aldehyde and 5-hydroxyconiferyl aldehyde (S channel). The experimentally validated channeling hypothesis permits 19 different topological configurations (Figure 3A) that satisfy the following constraints. First, at least one edge must be leaving caffeyl aldehyde and feruloyl CoA, and at least one edge must be entering coniferyl alcohol and 5-hydroxyconiferyl aldehyde; otherwise mass would unduly accumulate in intermediate pools. Second, if coniferyl aldehyde can be produced by a free CCR1 and/or COMT, it must also be consumed by a free enzyme, thereby decreasing the metabolic burden that would otherwise be imposed on the cell. For reasons that will be explained below, we also consider for each topological configuration various crosstalk patterns between the CCR2/COMT and CCoAOMT/CCR1 pathways. Each pattern is composed of documented or postulated mechanisms of metabolic regulation (activation or inhibition) (Figure 3B). The specific combinations of topological configurations and crosstalk patterns lead to hundreds of different designs, which were analyzed and compared (see Figure S4).

For each design, we first constructed 100,000 Generalized Mass Action (GMA) models by randomly sampling loosely-constrained parameter combinations from a parameter space that was deemed biologically realistic. A notable feature of this approach was that the parameter space was not only constrained at the level of individual parameters (*e.g.* kinetic orders), but also at the level of steady-state fluxes. For instance, the ratio of fluxes leading to S and G lignin was fixed at a value observed in the wild-type *Medicago* species (see Materials and Methods and Text S1 for details). Once all parameters for a given GMA model instantiation were specified, we determined steady-state fluxes under conditions that mimic CCoAOMT and COMT down-regulated alfalfa lines as well as *ccr1* and *ccr2 M. truncatula* mutant lines and computed the S/G ratios for which we had experimental data. We declared a model as *valid* if it yielded quantitatively and qualitatively correct results for both transgenic alfalfa and *M. truncatula* plants (see Materials and Methods). To assess the robustness of a design to parametric perturbations, we defined *Q* as the total number of valid model instantiations.

### Channels are necessary but not sufficient

**Figure 4:**
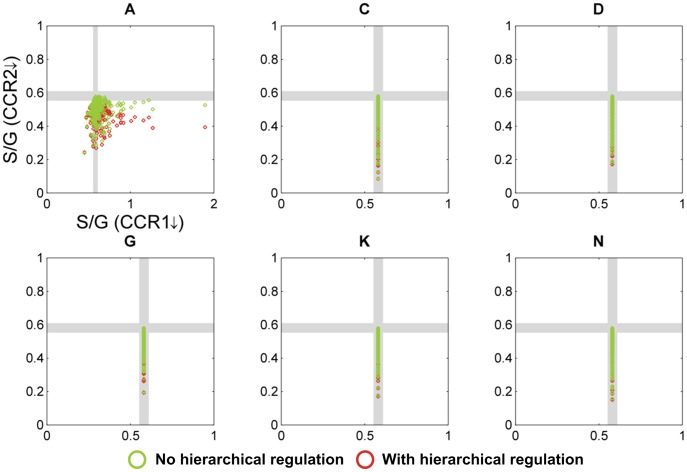
Simulation results for pathway designs without crosstalk. Each of the 6 panels represents one topological configuration (as indicated with labels (A, C, D, G, K, N) corresponding to model designs in Figure 3) with at least one randomly parameterized S-system model that yields quantitatively correct predictions of S/G ratios for both CCoAOMT and COMT down-regulated alfalfa plants. Each open circle refers to the S/G ratio of CCR1 and CCR2 in a *M. truncatula* knockout mutant, as predicted by one randomly parameterized S-system model; its color indicates the type of regulation. The gray strips denote regions within 5% of the wild-type level; model predictions within these strips are considered essentially the same as wild-type. Qualitatively correct predictions should fall into the northwest quadrant. It is evident that not a single model instantiation is admissible. The total number of randomly parameterized model instantiations per panel was 10^5^.

As a reasonable baseline, we first assumed the absence of crosstalk between the CCR2/COMT and CCoAOMT/CCR1 pathways (Figure 4). Of all possible topological configurations lacking crosstalk, only six had at least one parameter combination that yielded quantitatively correct predictions of S/G ratios for CCoAOMT and COMT down-regulated alfalfa plants. Supporting our previous findings [12], all six configurations include either one or both channels, suggesting that the channels are necessary. In other words, the pathway models are consistent with the observed changes in the S/G ratios of CCoAOMT and COMT down-regulated alfalfa plants only if at least one channel is present.

To assess these initially feasible parameter combinations further, we used the models with these parameter values to predict the S/G ratios for *ccr1* and *ccr2* knockout mutants. The *M. truncatula* lines harboring transposon insertions in *CCR1* and *CCR2* show a corresponding reduction in CCR1 and CCR2 activity, and their S/G ratio is decreased or increased, respectively, compared to the wild-type level [7]. Moreover, the activities of CCR1 and CCoAOMT, as well as their mRNA transcripts and proteins, are increased in the *ccr2* knockout mutant, indicating that part of their activation might be processed through a hierarchical control of gene expression [14]; see also Figure S4.

Figure 4 shows simulation results for those topological configurations where at least one out of 100,000 randomly parameterized models yielded quantitatively correct predictions of S/G ratios for both CCoAOMT and COMT down-regulated alfalfa plants. In these plots, a model is valid only if its predicted S/G ratios for *ccr1* and *ccr2* knockout mutants fall within the northwest quadrant.

In the case of no hierarchical regulation, *i.e.*, the *ccr2* mutant exhibits only reduced CCR2 activity, some model instantiations from configuration A showed a decreased S/G ratio for the *ccr1* knockout mutant, but not a single case exhibited an increased S/G ratio for the *ccr2* knockout mutant. This outcome did not improve much when hierarchical regulation was considered: not one of the 1.9 million model instantiations from the 19 possible configurations yielded qualitatively acceptable predictions for both *ccr1* and *ccr2* knockout mutants. These findings indicate that the S and G channels alone are not sufficient to explain all available transgenic data, and that some type of crosstalk is highly likely to occur between the CCR2/COMT and CCoAOMT/CCR1 pathways.

### Crosstalk between the CCR2/COMT and CCoAOMT/CCR1 pathways

One potential source of crosstalk between the CCR2/COMT and CCoAOMT/CCR1 pathways is substrate competition. CCR1/2 converts hydroxycinnamoyl CoA esters to their corresponding cinnamyl aldehydes, whereas CCoAOMT and COMT together complete the methylation of the aromatic C_3_ and C_5_ positions of the aldehydes and alcohols (Figure 1). All these enzymes are known to be multi-functional, acting upon multiple substrates with distinct catalytic efficiency. Because of their promiscuous nature, different substrates compete with each other if the supply of enzyme is limited. As a consequence, the enzymatic conversion of one substrate is effectively subjected to competitive inhibition by another substrate, and vice versa. This type of cross-inhibition is not necessarily equally strong in both directions because a promiscuous enzyme often displays preference for some substrates over others.

In the case of lignin biosynthesis, two regulatory mechanisms could arise from substrate competition. First, recombinant *Medicago* CCR2 exhibits similar *k*_cat_/*K*_M_ values for caffeoyl CoA (0.49 µM^−1^•min^−1^) and feruloyl CoA (0.40 µM^−1^•min^−1^) [7], suggesting that the CCR2-mediated conversion of caffeoyl CoA to caffeyl aldehyde in *Medicago* might be competitively inhibited by feruloyl CoA (Figure 3B; Mechanism 1). Furthermore, CCR2 is inhibited by feruloyl CoA at a concentration above 20 µM [7]. Conversely, it is highly unlikely that the CCR1-mediated conversion of feruloyl CoA to coniferyl aldehyde is significantly affected by caffeoyl CoA, because CCR1 has a *k*_cat_/*K*_M_ value for caffeoyl CoA (0.019 µM^−1^•min^−1^) that is 60 times lower than that for feruloyl CoA (1.14 µM^−1^•min^−1^) [7].

Second, the methylation of caffeoyl CoA by the combined activity of COMT and CCoAOMT may be subject to weak competitive inhibition by caffeyl aldehyde (Figure 3B; Mechanism 2). This assumption is based on the following observation. Although the combined *O*-methyltransferase (OMT) activity against caffeoyl CoA in extracts from internodes 6 to 8 of CCoAOMT-down-regulated alfalfa was reduced by 4.2-fold compared with the empty vector control line, about ∼25% of OMT activity remained [15]. This activity is presumably associated with COMT, for which caffeyl aldehyde is the preferred substrate. Notably, both mechanisms are independent of each other and may work individually or collaboratively to establish crosstalk between the two channels, thereby leading to three different crosstalk patterns and 57 different designs.

In the case where only Mechanism 1 (Figure 3B) was incorporated in the design, we observed a substantial increase in the number of model instantiations showing a decreased S/G ratio for the *ccr1* knockout mutant (Figure S1). Yet, even when we accounted for the effect of hierarchical regulation, none of the models was capable of delivering a qualitatively correct change in the S/G ratio for the *ccr2* knockout mutant. This finding indicates that the experimentally inferred inhibition evidently exists but is not sufficient. Similarly, we found no valid models when Mechanism 2, either by itself or coupled with Mechanism 1, was employed (Figures S2 and S3). An explanation may be that, with caffeyl aldehyde inhibiting the 3-*O*-methylation of caffeoyl CoA, knocking down CCR2 activity will consistently lead to a deregulation of CCoAOMT by caffeyl aldehyde, thereby increasing the flux to G lignin and reducing the S/G ratio.

### Is caffeyl aldehyde an activator of CCoAOMT?

**Figure 5:**
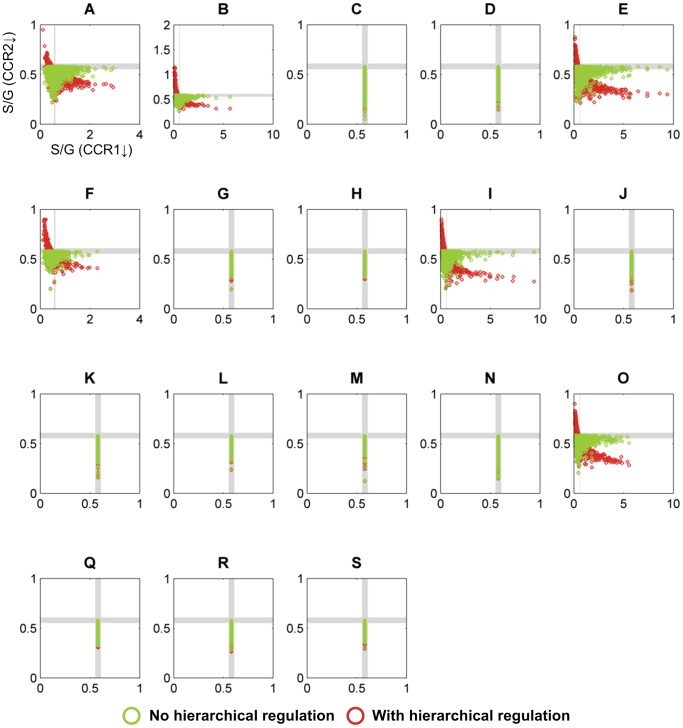
Simulation results for pathway designs using only Mechanism 3. See Figure 3B for the structure of this mechanism and the legend of Figure 4 for more information on details shown. In contrast to the results in Figure 4, the pathway designs analyzed here permit numerous admissible model instantiations (topologies A, B, E, F, I, and O), which fall into the northwest quadrant. The total number of randomly parameterized model instantiations per panel was 10^5^.

**Figure 6:**
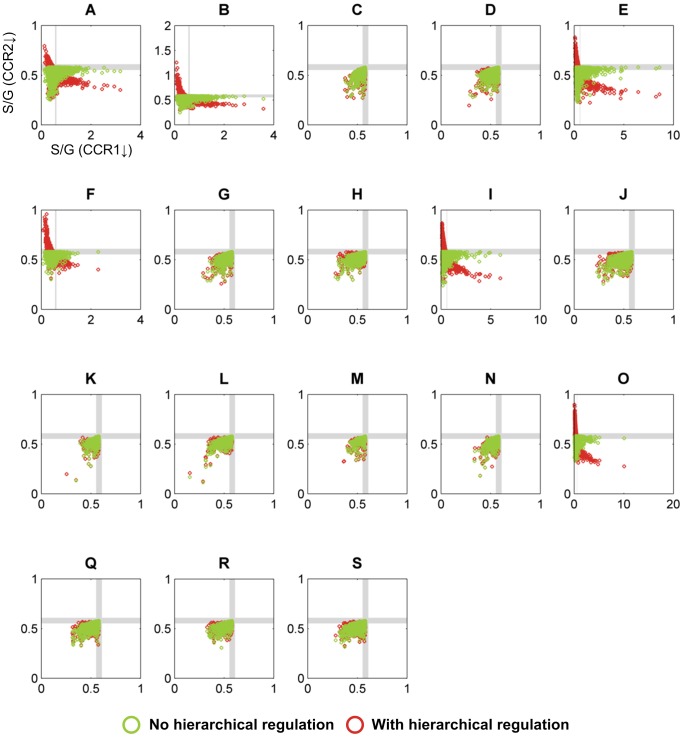
Simulation results for pathway designs that contain Mechanisms 1 and 3 simultaneously. See Figure 3B for the structure of these mechanisms and the legend of Figure 4 for more information on details shown. Similar to the results in Figure 5, the pathway designs analyzed here permit numerous admissible model instantiations (topologies A, B, E, F, I, and O), which fall into the northwest quadrant. The total number of randomly parameterized model instantiations per panel was 10^5^.

One could surmise that the 3-*O*-methylation of caffeoyl CoA, for which CCoAOMT is the primary enzyme, is actually activated by caffeyl aldehyde. This conjecture is based on the following argument. When the production of S lignin is compromised due to a knockout of *ccr2*, the only way of raising the S/G ratio beyond its wild-type level is to further reduce the flux through the CCoAOMT/CCR1 pathway, which can be accomplished if CCoAOMT is activated by caffeyl aldehyde. The simulation results using this type of postulated mechanism, either by itself (Figure 5) or coupled with the documented inhibition of CCR2 by feruloyl CoA (Figure 6), are very intriguing: For each crosstalk pattern where millions of randomly parameterized models were generated, we found thousands of valid instantiations that yielded quantitatively and qualitatively correct predictions for both transgenic alfalfa and *M. truncatula* plants. Perhaps more surprisingly, only six topological configurations (A, B, E, F, I, O) had at least one valid model (*Q*>0; see Materials and Methods). To ensure that this result was not due to the use of overly restrictive thresholds, we relaxed the criteria and found more parameter combinations that qualified. Nevertheless, the same six topological configurations always passed the screening test by a wide margin (Table S1). Collectively, these findings suggested that this activation mechanism, acting alone or with the inhibition of CCR2 by feruloyl CoA, is necessary for consistency with the *ccr1* and *ccr2* knockout data. This conclusion immediately translated into a targeted hypothesis that was independent of specific parameter choices and readily testable by experiment.

### The hypothesized activation is not supported by experimental data

**Figure 7:**
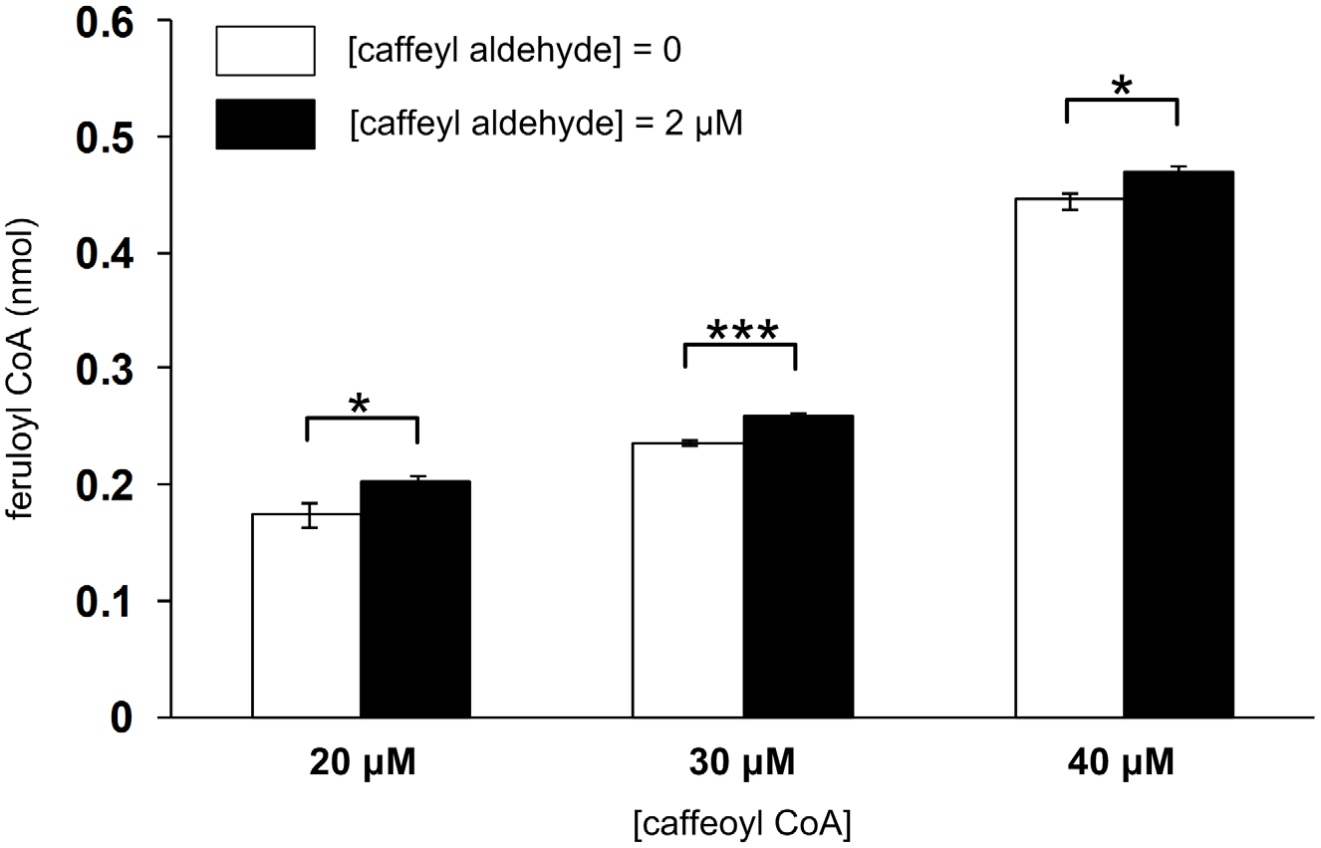
Experimental data indicating that the activation of CCoAOMT-mediated methylation of caffeoyl CoA by caffeyl aldehyde, while statistically significant, is too small to be biologically significant. Error bars, mean ± s.d.; ***p<0.001, *p<0.05 by Student's *t*-test; *n* = 3.

To examine whether caffeyl aldehyde indeed activates CCoAOMT, we expressed alfalfa CCoAOMT in *Escherichia coli* and assayed the purified recombinant enzyme with caffeoyl CoA as substrate and caffeyl aldehyde as the putative activator. As shown in Figure 7, the CCoAOMT activity increased by 16% at 2 µM of caffeyl aldehyde and 20 µM of caffeoyl CoA; at higher substrate concentrations (*i.e.*, 30 and 40 µM of caffeoyl CoA), the increase in mean CCoAOMT activity became less. Assays using lower concentrations of the substrate caffeoyl CoA (2, 4, 5 and 10 µM) and the putative activator caffeyl aldehyde (0.5, 1, 2 and 4 µM) showed no increase in CCoAOMT activity compared to the reaction without caffeyl aldehyde (data no shown). The maximal activation achieved *in vitro* was only 16%, which may not be biologically significant.

### Analysis of caffeyl aldehyde as a dual inhibitor of two 3-*O*-methylation reactions

Since a direct activation of CCoAOMT by caffeyl aldehyde was not observed with recombinant enzymes, we tested other regulatory mechanisms by themselves and in combination with known mechanisms. According to one possible mechanism, based again on the concept of substrate competition, caffeoyl CoA could be a competitive inhibitor for the 3-*O*-methylation of caffeyl aldehyde (Figure 3B; Mechanism 4). This proposal agrees with the fact that CCoAOMT may contribute up to ∼10% of the methylation reaction in alfalfa [15]. In addition, evidence in ryegrass (*Lolium perenne*) points to the possibility of COMT being inhibited by different substrates, such as caffeyl aldehyde and 5-hydroxyconiferyl aldehyde [16]. Interestingly, substrate inhibition by caffeyl alcohol and 5-hydroxyconiferyl alcohol has also been observed in *Selagniella moellendorffii* COMT [9]. Thus, we hypothesized that COMT might be inhibited by caffeyl aldehyde (Figure 3B; Mechanism 5) in *Medicago* as well; direct evidence supporting this hypothesis in *Medicago* remains to be determined.

**Figure 8:**
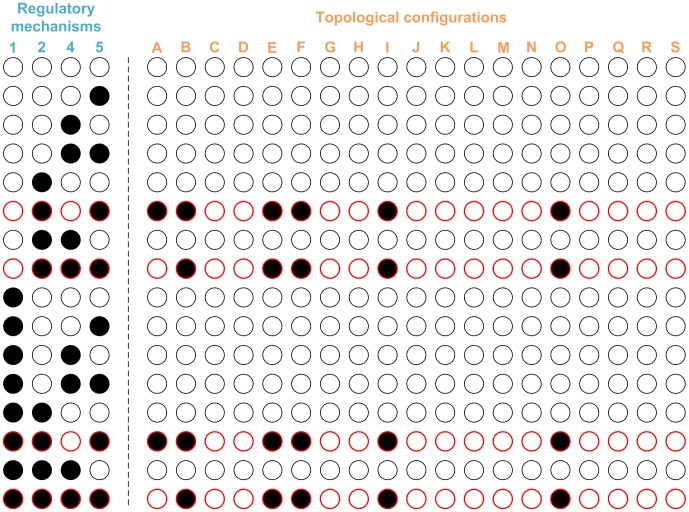
Summary of simulation results from 304 designs. Each row corresponds to one crosstalk pattern, whereas each column in the table to the right of dashed line corresponds to one topological configuration. A design is represented by a filled circle if at least one of the 10^5^ randomly parameterized model instantiations is valid. Empty circles thus refer to designs that are incongruent with observations. Rows highlighted in red contain at least one topological configuration with valid model instantiations.

In total, there are 2^4^ = 16 different crosstalk patterns that can result from the combination of four independent regulatory mechanisms (Figure 3B; Mechanisms 1, 2, 4 and 5). However, only four of them, when combined with the same six topological configurations (A, B, E, F, I and O) that were identified previously (cf. Figures 5 and 6), gave rise to designs with at least one valid model instantiation (Figure 8). Interestingly, all these crosstalk patterns require that caffeyl aldehyde is an inhibitor of the 3-*O*-methylation of both caffeoyl CoA and itself (Figure 3B; Mechanisms 2 and 5), providing computational evidence that this synergy between the two seemingly unrelated mechanisms is necessary for consistency with the *ccr1* and *ccr2* knockout data. Indeed, with respect to the *ccr2* knockout, such a combination of two inhibition mechanisms appears to have a similar ultimate effect as a single activation mechanism (see Discussion section).

**Figure 9:**
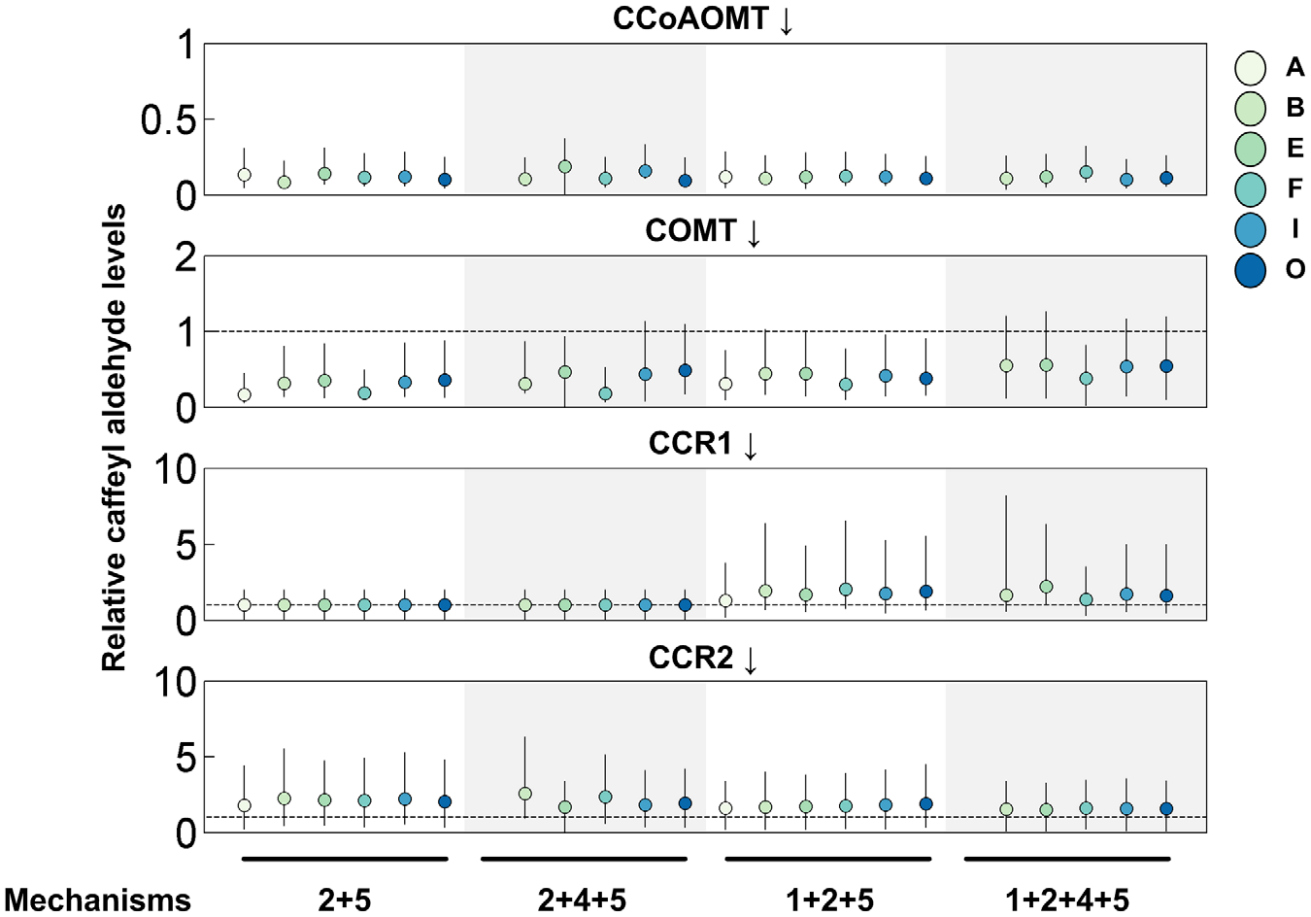
Relative levels of caffeyl aldehyde (compared to wild-type values) in simulations of four down-regulated lines. Each panel is shaded to highlight the results from four different crosstalk patterns. These patterns, when combined with specific topological configurations, give rise to designs with valid model instantiations (*cf.* rows with red circles in Figure 8). In contrast to the other three perturbation schemes, where all four crosstalk patterns (and their corresponding designs) exhibit similar responses regarding the caffeyl aldehyde level, knocking out *ccr1* is associated with a higher caffeyl aldehyde level only for the two crosstalk patterns including Mechanism 1. The circles, colored according to topological configuration, are the medians, and the error bars represent interquartile ranges. The dashed line in each panel, if present, denotes the wild-type level of caffeyl aldehyde.

Inspecting the crosstalk patterns giving rise to at least one design with valid model instantiations (rows colored in red in Figure 8), one might surmise that caffeyl aldehyde would accumulate to an unduly high level, because Mechanism 5, which is employed in all these patterns, reflects substrate inhibition of COMT by caffeyl aldehyde. To examine the validity of this inference, we checked, for all designs with valid model instantiations, the predicted changes in caffeyl aldehyde under conditions that mimic the down-regulation of four lignin biosynthetic enzymes. As shown in Figure 9, it appears that down-regulation of CCoAOMT or COMT is consistently associated with a lower caffeyl aldehyde level compared with wild type, regardless of the crosstalk pattern being considered. Similarly, knocking out *ccr2* consistently raises the caffeyl aldehyde level in all crosstalk patterns examined. However, in the case of the *ccr1* knockout mutant, the results are mixed in a sense that some crosstalk patterns are associated with significantly higher caffeyl aldehyde levels, whereas others are associated with only modest changes. Interestingly, both crosstalk patterns suffering from an undue accumulation of caffeyl aldehyde contain Mechanism 1. By contrast, this mechanism is absent from other patterns, which maintain a relatively stable caffeyl aldehyde level. This finding suggests that the control pattern in Mechanism 1 may disrupt the metabolic homeostasis via accumulation of caffeyl aldehyde when CCR1 drops below its normal level. As any cellular system is constantly afflicted by a variety of intrinsic and extrinsic noises, this type of fluctuation must be expected to occur frequently and spontaneously, suggesting that Mechanism 1 is disadvantageous.

### Robust designs are evolutionarily connected

Investigation of the six robust topological configurations, which contain at least one valid model instantiation, revealed interesting structural features of the pathway. In particular, the G lignin channel is common to all robust designs and thus may be considered critical for the proper functioning of the pathway, at least for the cases studied. The evolutionary conservation of such a feature, one may argue further, is not due to the fact that it cannot possibly be altered, but that this particular design can sustain maximally tolerable changes and variability in other features [17]. These arguments lead to an interesting follow-up question, namely: Are the robust topological configurations related in an evolutionary sense?

To address this question, we constructed a “topology graph” where each node corresponds to a topological configuration. Two nodes are connected if the corresponding topological configurations differ only by one edge. For instance, configurations A and B are directly linked to each other because the only difference between them is whether caffeyl aldehyde can be converted, via free COMT, to coniferyl aldehyde. In other words, moving from a node to its neighbor may be considered a singular evolutionary event where an enzyme's preferred mode of action is changed.

**Figure 10:**
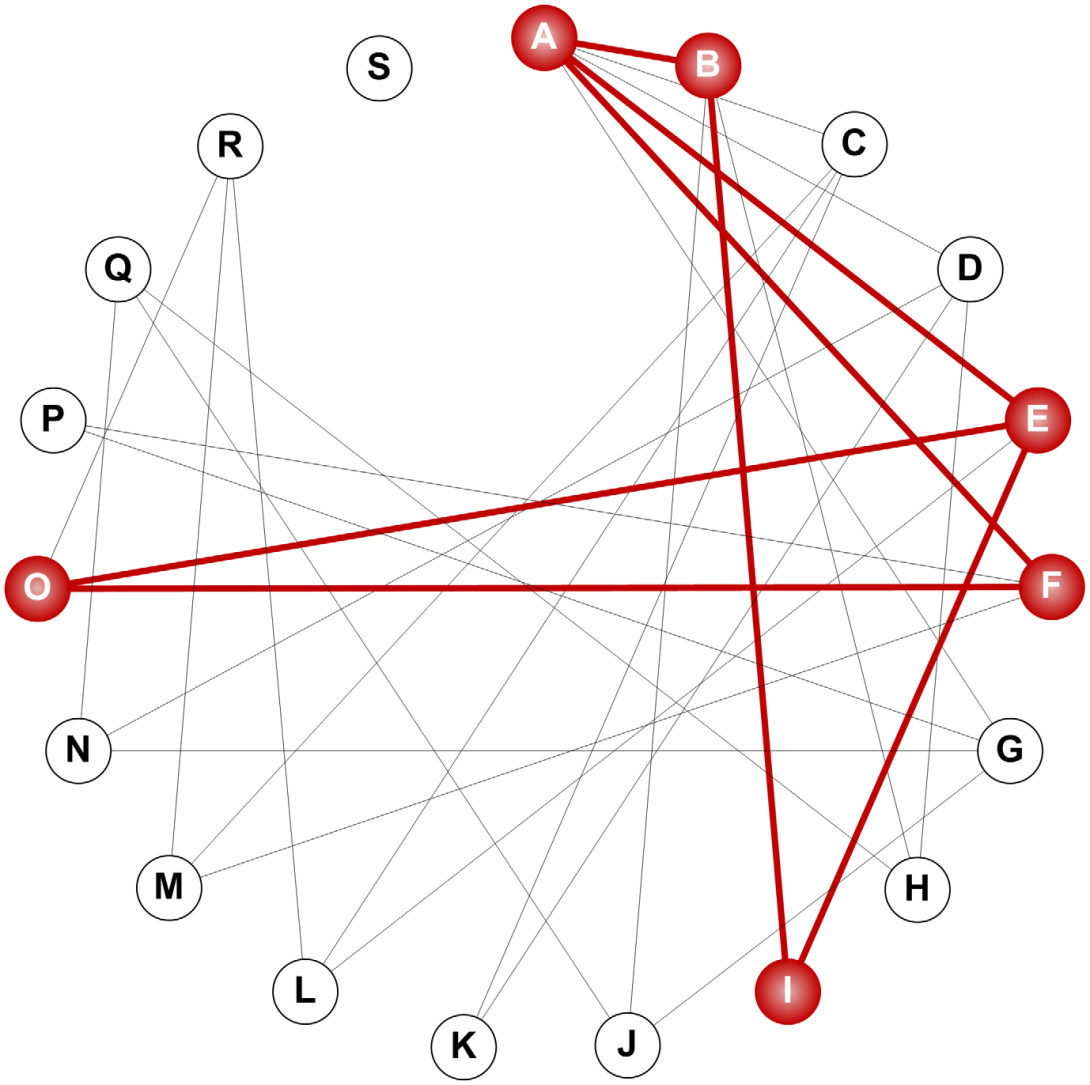
Robust configurations are evolutionarily connected. Each node represents a specific topological configuration (see Figure 3); two nodes are connected if the corresponding configurations differ only by one edge. The subgraph of all the robust configurations, colored in red, is connected, thereby indicating the potential of direct evolvability.

Two outcomes are possible for the structure of such a topology graph. First, the graph may be disconnected, that is, there exist pairs of topological configurations such that no evolutionary path (defined as a series of evolutionary events) connects one to the other. In the most extreme case, the graph would consist exclusively of isolated nodes. Second, the graph is fully connected, so that any pair of topological configurations is connected by at least one evolutionary path. As shown in Figure 10, the actual topology graph of the six robust configurations of lignin biosynthesis is indeed connected, and so is the graph of all configurations, except for design S. This interconnectedness can be interpreted as facilitating the evolvability of the system [17], because the gain or loss of specific features that are needed to produce phenotypically novel traits will be tolerated and survive during evolution if robustness is preserved. Of course, this evolutionary aspect, which was derived purely with computational means, will require additional analysis.

## Discussion

The spatial organization of cooperating enzymes, known as metabolic channeling, has long been recognized as an effective means of regulation in primary and secondary plant metabolism [18], [19], [20]. This channeling phenomenon involves the organization of enzymes into complexes and/or the co-localization of enzymes at the plasma membrane or on the surfaces of organelles, as was demonstrated for the two initial enzymes, L-phenylalanine ammonia-lyase (PAL) and cinnamate 4-hydroxylase (C4H), in the phenylpropanoid pathway [21], [22]. Interestingly, some complexes or interactions are persistent, while others are temporary. In fact, many of the component enzymes such as PAL may be operationally soluble and are therefore only facultatively channeled. Such short-lived or dynamic complexes, while being readily responsive to the metabolic status of the cell, are inherently difficult to study with existing or emerging experimental models.

Using the lignin biosynthetic pathway as a model system, we propose here a novel strategy for studying metabolic channeling in unprecedented detail. Specifically, we consider all possible modes of action for both the G lignin and S lignin channels, and these can be mapped into 19 different topological configurations (Figure 3A). Metabolic channeling is clearly not the only process that affects the functionality of this system, and it is therefore necessary to study control processes affecting a channeled system. In the present case, this control is potentially exerted by individual or combined mechanisms of crosstalk between the CCR2/COMT and CCoAOMT/CCR1 pathways (Figure 3B). Some of these were documented in the literature, while others were hypothesized. Taken together, a topological configuration and a specific crosstalk pattern constitute a design. We evaluated each design with or without consideration of non-allosteric or hierarchical regulation which could involve transcription, as well as a variety of non-transcriptional processes such as phosphorylation, methylation, and targeted degradation of proteins and mRNA.

Ideally, the comparative assessment of design features would be entirely symbolic and independent of specific parameter values. However, systems of a realistic size are rarely analyzable in such fashion. As a reasonable alternative, we analyzed the possible design space comprehensively with widely varying parameter values, which resulted in a computational analysis of millions of models from hundreds of designs. This analysis yielded several interesting findings.

Importantly, it predicted that CCoAOMT is directly or indirectly activated by caffeyl aldehyde. This piece of information by itself is essentially unbiased, but insufficient to explain the exact mechanism of regulation. Nevertheless, it offered a specifically targeted hypothesis and was therefore experimentally testable. However, the hypothesis of a direct activation was refuted by subsequent experiments using the recombinant *Medicago* CCoAOMT, which failed to provide evidence confirming the putative role of caffeyl aldehyde as an allosteric activator. It might still be possible that activation exists *in vivo*, but it seems more likely that the activation is indirect rather than direct.

As a possible mechanism, the design analysis suggested that caffeyl aldehyde inhibits the 3-*O*-methylation of both caffeoyl CoA and itself. Several lines of evidence, although not exclusively from *Medicago*, support this computational prediction. Most importantly, the same six topological configurations were identified in the indirect design analysis and in the initial analysis of a putative activation mechanism. However, the two most parsimonious mechanisms differ in their proposed control strategies. The original analysis suggested just one activation mechanism, while the second analysis proposed two inhibition mechanisms. To some degree, these two mechanisms have the same ultimate effect. If *ccr2* is knocked out, the flux entering the CCR2/COMT pathway and the subsequent synthesis of S lignin decline. The only possibility to increase the S/G ratio is to reduce the flux entering the CCoAOMT/CCR1 pathway. This task can be accomplished either through a diminished activation, as suggested for the single activation mechanism, or through an enhanced inhibition, as suggested for the dual-inhibition mechanism. The latter mechanism seems sufficient to restore consistency with the data, but it is of course possible that more complicated control patterns are present.

The computational analysis suggests that the G lignin channel is necessary for the system to respond correctly and robustly to certain genetic perturbations. By contrast, the S lignin channel appears to be dispensable. This theoretical deduction is indirectly in line with the fact that S lignin has arisen much later in the evolution of higher plants than G lignin [1]. It is also consistent with the observation that its formation, which in many plant species is dictated by F5H expression [23], [24], [25], is directly regulated by a secondary cell wall master switch NST1/SND1 and not by MYB58, a SND1-regulated transcription factor that can activate other lignin biosynthetic genes [26]. It could also be possible that S lignin, which is specifically involved in the pathogen defense of some plants [27], was relatively recently recruited for lignin biosynthesis and thus may not be essential for plant growth. Evidence supporting this postulate includes an *Arabidopsis* NST1/SND1 double knockout mutant that shows a complete suppression of secondary cell wall thickening in woody tissues, including interfascicular fibers and secondary xylem, but otherwise grows quite well as compared to the wild-type plants [28].

Within an evolutionary context, the multiplicity of robust solutions can be represented with a graph representation that connects any two (robust) topological configurations differing by a single edge. This graph is reminiscent of the “neutral network” concept that was initially proposed in genotype-phenotype models for RNA secondary structures [29] and protein folds [30], but also more recently extended to Boolean models for gene regulatory networks [31]. In the case of proteins, neutral networks are defined as sets of amino acid sequences that are connected by single-mutation neighbors and that map into the same tertiary structure. Such degeneracy of the mapping from genotype to phenotype allows a neutral drift in genotypic space, which is critical for accessing adjacent neutral networks with novel phenotypes that may confer higher fitness to the cells. As of yet, it is unclear whether individual plants within a *Medicago* population use the same or different designs, or whether the response to selected perturbations is an adequate phenotypic feature. Further investigation of the protein-protein interactions between lignin biosynthetic enzymes is thus necessary to confirm that a G lignin channel is indeed necessary for optimal functioning.

The work in this article describes a novel computational approach that shows promise in deciphering the principles of channel assembly in a biosynthetic pathway when relevant information is limited. It also provides a clear direction in which to proceed with more targeted experiments. Beyond the application described here, the proposed strategy might be beneficial in entirely different biological contexts, such as gene regulatory and signaling networks, where the task is to analyze how information flow is controlled by the spatial organization of molecules in the cell.

## Materials and Methods

### Model equations in GMA format

Since the two metabolic channels of interest are assumed to affect only the relative amounts of G and S lignin, the analysis is restricted to those critical steps within the lignin biosynthetic pathway system that govern the flow of material either toward G or S (Figure 2). For each possible design, we first formulate the corresponding generalized mass action (GMA) model [32], [33] in a symbolic format, where each intermediate is represented by a dependent variable and each enzymatic process by a product of power-law functions. An important reason for this choice of a modeling format is that it is mathematically sound and minimally biased, because it does not require the *a priori* specification of a biological mechanism [34]. The model contains either six or seven dependent variables, depending on whether coniferyl aldehyde is explicitly included, and 10 to 16 distinct power-law terms, depending on the topology in a specific design. Also, there are six independent variables, each of them representing the extractable activity of an enzyme. Each power-law term contains two different types of parameters: a non-negative rate constant *γ_i_* that represents the turnover of reaction *i*, and kinetic orders *f_i,j_*, each of which characterizes the effect of a variable *X_j_* on a reaction *i*. A kinetic order can take any real value, and the sign of its value has a directly interpretable meaning: a positive value indicates an activating effect, a negative value an inhibiting effect, and zero no effect.

As a typical example, the differential equation for caffeoyl CoA, defined as *X*_1_, has two representations (*cf.* Figure S4B and [33] for further details):
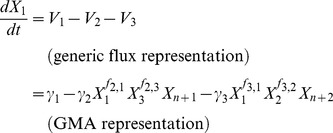
(1)The variables *V_i_* in the first equation represent the reaction rates, or fluxes, in a generic format. In the corresponding GMA model, these fluxes are specifically modeled as products of power-law functions of dependent and independent variables *X_i_*_._ Aside from *X*_1_, the fluxes contain two other dependent variables, *X*_2_ (caffeyl aldehyde) and *X*_3_ (feruloyl CoA), as well as two independent variables, *X*_n+1_ (CCR2) and *X*_n+2_ (CCoAOMT). In GMA representations, *n* typically denotes the number of dependent variables, so that *n*+1 and *n*+2 refer to the first two independent variables. At the steady state, the derivative on the left-hand side becomes zero, thereby turning the differential equation into a linear equation of fluxes (*V_iS_*; where *S* denotes the steady state)
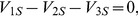
(2)with the following constraints
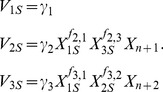
(3)


Notably, *X*_3_ and *X*_2_ are included in the power-low representations of *V*_2_ and *V*_3_, respectively, because they potentially modulate the two consuming fluxes of *X*_1_. Applying the rules for kinetic orders described above, we can immediately impose bounds on the values of *f*_2,3_ and *f*_3,2_ for different regulatory mechanisms (Figure 3B). For instance, modeling Mechanism 1 requires the following constraints,
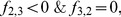
(4)because *X*_3_ is considered an inhibitor, so that *f*_2,3_<0, while *X*_2_ has no influence on the degradation of *X*_1_ through reaction 3 in this design, so that *f*_3,2_ = 0, which in effect eliminates the factor 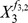 from the term on the far right. By convention, all independent variables have a kinetic order of 1.

### Sampling of steady-state fluxes

Determination of all parameters in a GMA model, including kinetic orders and rate constants, is required prior to most simulation tasks. While numerous methods have been developed over the years, parameter estimation is seldom straightforward as each pathway and each dataset has its own challenges [35]. For the lignin pathway in *Medicago*, very little information is available on exact concentrations of intermediates or fluxes through the pathway; in fact, many metabolites *in vivo* are below detection level with standard HPLC [36].

To address this issue of insufficient data, we sample parameter values from relatively wide, biologically realistic ranges. The procedure involves the following steps. First, we sample the steady-state fluxes *V_iS_* from a set *P* in an *m*-dimensional vector space (**R** *^m^*), where *m* equals the number of reactions. The sampled steady-state fluxes can be thought of as possible representatives of a wild-type *Medicago* species, while *P* defines the boundaries within which the system is able to operate. Biologically, these boundaries are given by many linear equality or inequality constraints with physiological meaning, such as the reaction stoichiometry (*e.g.* Eq. (2)), the ratio of S to G lignin in a mature stem internode of wild-type alfalfa, and the degree of reversibility of individual reactions (see Text S1 for further information). Mathematically, *P* is a bounded polyhedron (or polytope) and therefore has a concise parametric description
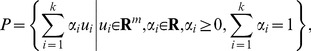
(5)where the *m*-dimensional vectors *u_i_* can be identified using first principles [37]; in a different context, the vectors *u_i_* have been called “extreme pathways” [38].

### Steady-state equations in S-system format

Once a set of steady-state reaction rates is randomly generated, we sample kinetic orders (*f_i,j_*) from their respective ranges (Table S2), which are chosen based on the unique role of each kinetic order. Even with this information, the lack of concentration data from a wild-type *Medicago* species remains an issue that needs to be solved. To this end, we perform two transformations. First, we define a normalization of variables by replacing *X_i_* with *Y_i_*≡*X_i_*/*X_iS_*, where *X_iS_* are the unknown steady-state levels of *X_i_* in wild type. As an example, the differential equation for caffeoyl CoA assumes the form
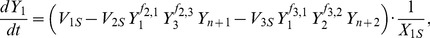
(6)where *V_iS_* are the steady-state reaction rates sampled from *P*. This representation is well suited for the current analysis because the exact values of *X_iS_* become irrelevant once all the equations are set to zero, that is, at a wild-type or perturbed steady state (*cf.* Figure S4C). Second, after all parameters for a given GMA model instantiation are specified, we derive the corresponding S-system equations with straightforward mathematical manipulations that do not require any additional biological information [33; Chapter 3]. At the steady state, GMA and S-system models are equivalent, but they offer different advantages for further analyses. In particular, S-system differential equations, despite being intrinsically nonlinear, become linear at the steady state after a logarithmic transformation, thereby facilitating the computation of secondary steady-state features and bypassing the time-consuming numerical integration that is otherwise required for assessing nonlinear models. Given this convenient feature, we are able to obtain, in a very efficient manner, estimates of steady-state fluxes under conditions that mimic the two transgenic alfalfa lines and two *M. truncatula* mutant lines; we can also easily compute the S/G ratios for which we had experimental data.

### Simulation of knock-down experiments

Down-regulation of specific lignin biosynthetic enzymes is simulated by setting the corresponding normalized independent variables *Y_i_* to values between 0 and 1 that represent the degree of down-regulation, and solving the steady-state equations. In cases where hierarchical regulation might be effective, such as in *ccr2* knockout mutants, all affected *Y_i_* are given values that mirror the specific changes in activities (*cf.* Figure S4D). The Parallel Computing Toolbox™ in MATLAB (version R2009b, The MathWorks, Natick, MA) was used to divide the simulation job among multiple cores for speedup.

Not all models behaved properly during simulation, and some ill-behaved models were excluded from further analysis. These were defined, arbitrarily, as models that showed a more than 1000-fold increase or decrease in any dependent variable during any simulation. Further, a properly behaved parameter set was deemed valid if the following criteria were met:

1. Quantitative correctness for simulations of CCoAOMT and COMT down-regulation, which was defined as a mean squared difference between the predicted and observed S/G ratios of less than 0.01 in these two cases.
2. Qualitative correctness for the simulations of *ccr1* and *ccr2* knockout mutants. Specifically, the predicted S/G ratio must show a decrease of more than 5% for *ccr1* (or an increase of more than 5% for *ccr2*), compared to the wild-type value.

These criteria for success of a model instantiation were initially determined in an *ad hoc* fashion: We applied more lenient, qualitative criteria to the predictions of S/G ratios for *ccr1* and *ccr2* knockout mutants because the experimental data were only available in *Medicago truncatula*, but not in our model organism alfalfa (the S/G ratio of every model instantiation was set to the experimentally determined value for the sixth internode of a wild-type alfalfa plant). However, when we relaxed the criteria for screening the predictions of S/G ratios for CCoAOMT and COMT down-regulated lines to allow a percentage error as large as 25%, we obtained the exact same set of pathway designs that are consistent with the data, suggesting that the main conclusions are quite robust to the choice of thresholds.

### Expression of alfalfa CCoAOMT in *E. coli*

The cloning of the alfalfa CCoAOMT cDNA into the expression vector pET15b was as described previously [15]. *E. coli* Rosetta strains containing the constructed plasmid were cultured at 37°C until OD_600_ reached 0.6–0.7, and protein expression was then induced by adding isopropyl 1-thio β-galactopyranoside (IPTG) at a final concentration of 0.5 mM, followed by 3 h incubation at the same temperature. Cell pellets from 25 ml induced medium were harvested and frozen at −80°C for further use. Induced cell pellets were thawed at room temperature, resuspended in 1.2 ml of extraction-washing buffer (10 mM imidazole, 50 mM Tris-HCl pH 8.0, 500 mM NaCl, 10% glycerol and 10 mM β-mercaptoethanol), and sonicated three times for 20 s. Supernatants were recovered after centrifugation (16,000×*g*), and incubated at 4°C for 30 min with equilibrated Ni-NTA beads (Qiagen, Germantown, MD) under constant inversion to allow the His-tag protein to bind to the beads. The beads were washed three times with 1 ml of extraction-washing buffer, and the target protein was eluted with 250 µl of elution solution (250 mM imidazole, 50 mM Tris-HCl buffer pH 8.0, 500 mM NaCl, 10% glycerol and 10 mM β-mercaptoethanol). The concentration of the eluted target protein was determined using the BioRad protein assay (BioRad, Hercules, CA) and its purity was verified by SDS-PAGE.

### Materials and enzyme activity assays

Caffeoyl CoA for the enzyme assays, and feruloyl CoA for the calibration curve, were synthesized as described previously [39]. Caffeyl aldehyde was synthesized as described by Chen et al. [40]. Pure recombinant CCoAOMT enzyme (100 ng) was incubated at 30°C for 20 min with 60 mM sodium phosphate buffer pH 7.5, 200 µM S-adenosyl methionine, 600 µM MgCl_2_ and 2 mM dithiothreitol. The substrate (caffeoyl CoA) concentration was 20, 30 or 40 µM and the putative activator (caffeyl aldehyde) concentration was 0, 2, 5 or 10 µM. Since caffeyl aldehyde was in dimethyl sulfoxide solution, the final concentration of dimethyl sulfoxide in the reaction was 4% and the final volume of the reaction was 50 µl. The reactions were stopped by adding 10 µl of 24% w/v trichloroacetic acid. Reaction products were analyzed by reverse-phase HPLC on a C18 column (Spherisorb 5 µ ODS2, Waters, Milford, MA) in a step gradient using 1% phosphoric acid in water as solvent A and acetonitrile as solvent B. Calibration curves were constructed with authentic standard of the product feruloyl CoA. Activity assays using lower concentrations of the substrate caffeoyl CoA (2, 4, 5 and 10 µM) and the putative activator caffeyl aldehyde (0.5, 1, 2 and 4 µM) were performed using a sensitive radioactive assay method as described previously [15].

## Supporting Information

Figure S1 **Simulation results for CCR1 and CCR2 down-regulation using only Mechanism 1.** As with Figures 3 and 4 in the main text, only topological configurations with at least one model showing quantitatively correct predictions for both CCoAOMT and COMT down-regulation are plotted.
(TIF)

Figure S2 **Simulation results for CCR1 and CCR2 down-regulation using only Mechanism 2.** See legend of Figure S1 for more details.
(TIF)

Figure S3 **Simulation results for CCR1 and CCR2 down-regulation using Mechanisms 1 and 2.** See legend of Figure S1 for more details.
(TIF)

Figure S4 **Model formulation and nomenclature.** (A) Definition of all dependent and independent variables for a system that implements configuration A in Figure 3; other designs are obtained by removing the appropriate fluxes. (B) Model equations in GMA format. (C) The equations for all steady-state solutions to the system of differential equations in (B) can be expressed using normalized variables. (D) Down-regulation of specific enzymes, represented as independent variables, is simulated by setting the corresponding *Y_i_* to values that represent the degree of down-regulation as observed in prior experiments.
(TIF)

Table S1 **Number of valid model instantiations as judged by two different robustness measures (** ***Q*** **and** ***Q*** **′).** Statistics with a non-zero value of *Q* or *Q*′ are marked in bold. The first number is from simulation with Mechanism 3 only, whereas the second number is from simulation with both Mechanisms 1 and 3.
(DOCX)

Table S2 **Upper and lower bounds for kinetic orders.**
(DOCX)

Text S1 **Details regarding the selection of target tissue and the definition of physicochemical constraints.** A supplementary text for Figure S4 is also included.
(DOCX)
