# Supplementary figures and images for: Functional Analysis of Metabolic Channeling and Regulation in Lignin Biosynthesis: A Computational Approach
Source: PLoS Comput Biol. 2012 Nov 8;8(11):e1002769. doi: 10.1371/journal.pcbi.1002769 (PMC3493464; doi:10.1371/journal.pcbi.1002769)

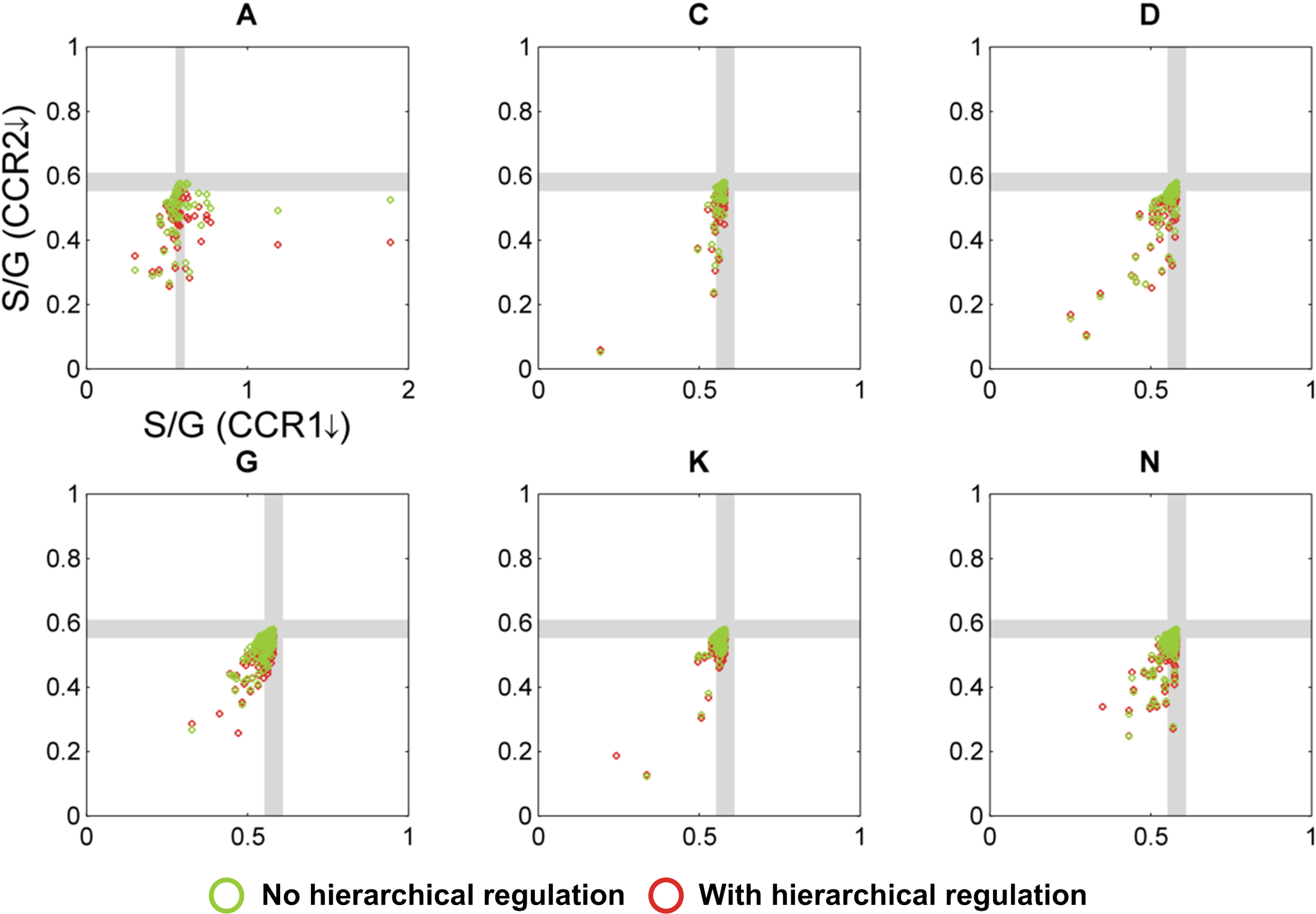

Supplement: Figure S1 — Simulation results for CCR1 and CCR2 down-regulation using only Mechanism 1. As with Figures 3 and 4 in the main text, only topological configurations with at least one model showing quantitatively correct predictions for both CCoAOMT and COMT down-regulation are plotted. (TIF) [file pcbi.1002769.s001.tif]

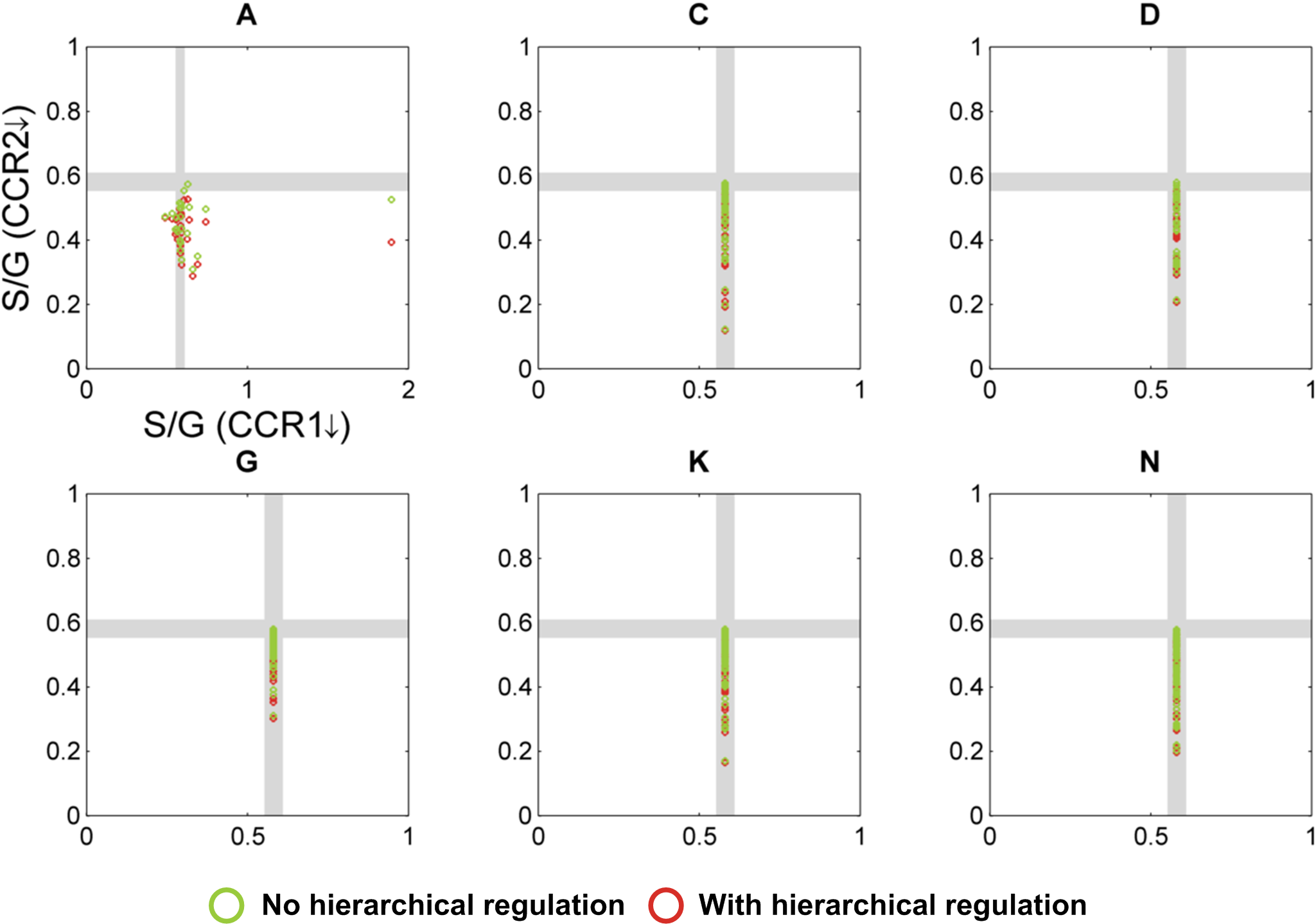

Supplement: Figure S2 — Simulation results for CCR1 and CCR2 down-regulation using only Mechanism 2. See legend of Figure S1 for more details. (TIF) [file pcbi.1002769.s002.tif]

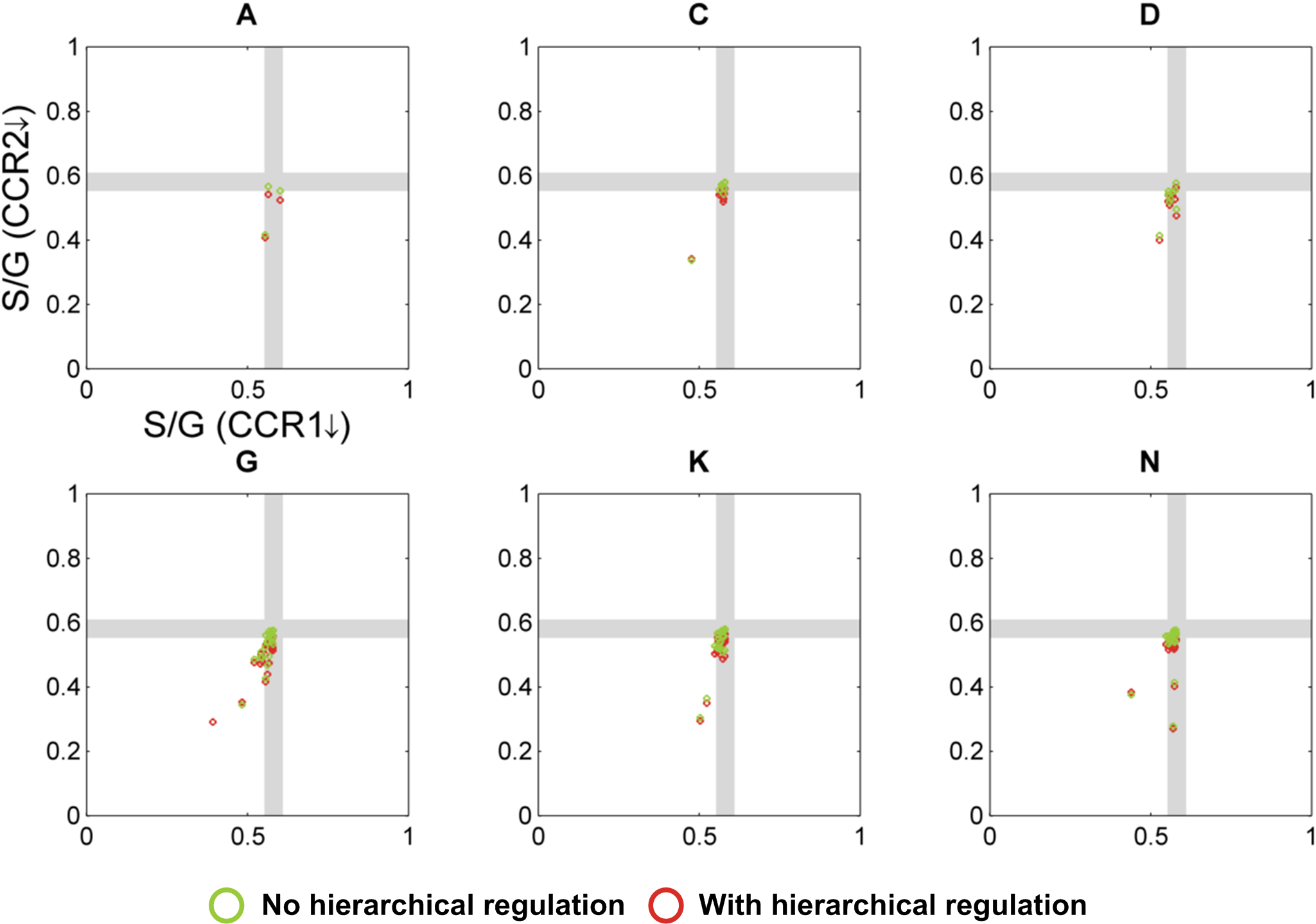

Supplement: Figure S3 — Simulation results for CCR1 and CCR2 down-regulation using Mechanisms 1 and 2. See legend of Figure S1 for more details. (TIF) [file pcbi.1002769.s003.tif]

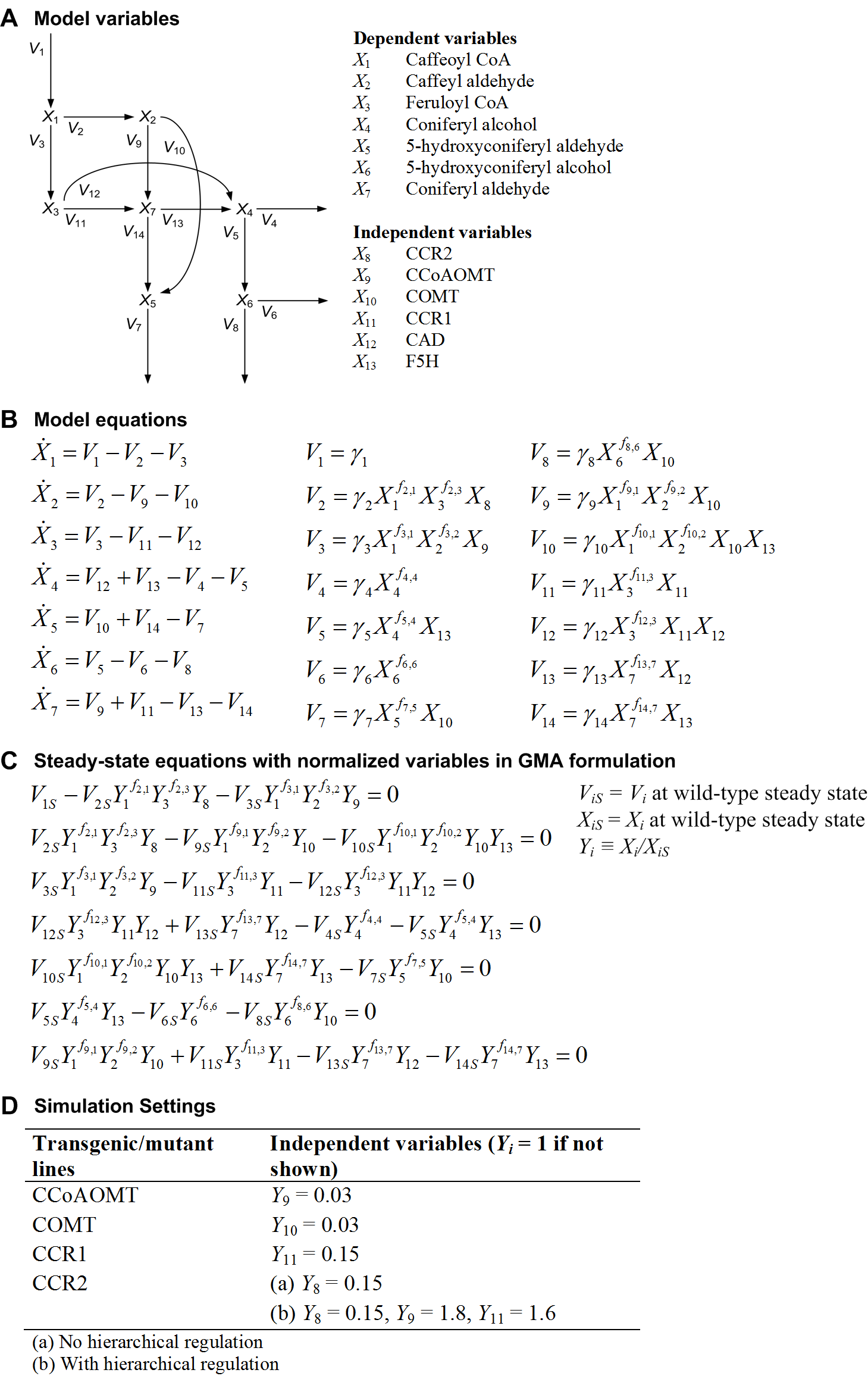

Supplement: Figure S4 — Model formulation and nomenclature. (A) Definition of all dependent and independent variables for a system that implements configuration A in Figure 3; other designs are obtained by removing the appropriate fluxes. (B) Model equations in GMA format. (C) The equations for all steady-state solutions to the system of differential equations in (B) can be expressed using normalized variables. (D) Down-regulation of specific enzymes, represented as independent variables, is simulated by setting the corresponding Yi to values that represent the degree of down-regulation as observed in prior experiments. (TIF) [file pcbi.1002769.s004.tif]
