# Supplementary material for: Functional Analysis of Metabolic Channeling and Regulation in Lignin Biosynthesis: A Computational Approach
Source: PLoS Comput Biol. 2012 Nov 8;8(11):e1002769. doi: 10.1371/journal.pcbi.1002769 (PMC3493464; doi:10.1371/journal.pcbi.1002769)
Supplement: Table S1 — Number of valid model instantiations as judged by two different robustness measures ( Q and Q ′). Statistics with a non-zero value of Q or Q′ are marked in bold. The first number is from simulation with Mechanism 3 only, whereas the second number is from simulation with both Mechanisms 1 and 3. (DOCX) [file pcbi.1002769.s005.docx]

Table S1**.** **Number of valid model instantiations as judged by two different robustness measures (*Q* and *Q*’).** Statistics with a non-zero value of *Q* or *Q*’ are marked in bold. The first number is from simulation with Mechanism 3 only, whereas the second number is from simulation with both Mechanisms 1 and 3.

| Configuration # | *Q* | *Q*’ |
| --- | --- | --- |
| A | **96/100** | **218/222** |
| B | **284/303** | **461/486** |
| C | 0/0 | 0/0 |
| D | 0/0 | 0/0 |
| E | **307/361** | **489/555** |
| F | **176/180** | **278/273** |
| G | 0/0 | 0/0 |
| H | 0/0 | 0**/4** |
| I | **431/422** | **626/649** |
| J | 0/0 | 0/0 |
| K | 0/0 | 0/0 |
| L | 0/0 | 0/0 |
| M | 0/0 | 0/0 |
| N | 0/0 | 0/0 |
| O | **381/447** | **619/662** |
| P | 0/0 | 0/0 |
| Q | 0/0 | 0/0 |
| R | 0/0 | 0/0 |
| S | 0/0 | 0/0 |

*Q* = # models showing a decrease of more than 5% and an increase of more than 5%, compared to the wild-type level, in simulations of CCR1 and CCR2 down-regulation, respectively.

*Q*’ = # models showing a decreased and an increased S/G ratio, compared to the wild-type level, in simulations of CCR1 and CCR2 down-regulation, respectively.
