# Supplementary material for: Functional Analysis of Metabolic Channeling and Regulation in Lignin Biosynthesis: A Computational Approach
Source: PLoS Comput Biol. 2012 Nov 8;8(11):e1002769. doi: 10.1371/journal.pcbi.1002769 (PMC3493464; doi:10.1371/journal.pcbi.1002769)
Supplement: Table S2 — Upper and lower bounds for kinetic orders. (DOCX) [file pcbi.1002769.s006.docx]

Table S2**.** **Upper and lower bounds for kinetic orders.**

| Kinetic order (*f*_enzyme, substrate/regulator_) | Lower bound | Upper bound |
| --- | --- | --- |
| *f*_CCR2, caffeoyl CoA_ | 0 | 2^a^ |
| *f*_CCoAOMT, caffeoyl CoA_ | 0 | 1^b^ |
| *f*_COMT, caffeyl aldehyde_ | 0 | 1 |
| *f*_COMT/F5H, caffeyl aldehyde_ | 0 | 1 |
| *f*_CCR1, feruloyl CoA_ | 0 | 1 |
| *f*_CCR1/CAD, feruloyl CoA_ | 0 | 1 |
| *f*_CAD, coniferyl aldehyde_ | 0 | 1 |
| *f*_F5H, coniferyl aldehyde_ | 0 | 1 |
| *f*_Tr, coniferyl alcohol_ | 1^c^ | 1 |
| *f*_F5H, coniferyl alcohol_ | 0 | 1 |
| *f*_COMT, 5-hydroxy coniferyl aldehyde_ | 0 | 1 |
| *f*_Tr, 5-hydroxy coniferyl alcohol_ | 1^c^ | 1 |
| *f*_COMT, 5-hydroxy coniferyl alcohol_ | 0 | 1 |
| all kinetic orders for activators | 0 | 2 |
| all kinetic orders for inhibitors | -2 | 0 |

^a^CCR2 shows positive cooperativity towards caffeoyl-CoA [2]

^b^A kinetic order of 0 corresponds to a Michaelis-Menten process where the enzyme is saturated, and a kinetic order of 1 describes the situation in which the substrate concentration is negligibly small compared to the Michaelis constant *K*_M_ [6].

^c^The transport process (Tr) is assumed to be first order
