# Supplementary material for: Functional Analysis of Metabolic Channeling and Regulation in Lignin Biosynthesis: A Computational Approach
Source: PLoS Comput Biol. 2012 Nov 8;8(11):e1002769. doi: 10.1371/journal.pcbi.1002769 (PMC3493464; doi:10.1371/journal.pcbi.1002769)
Supplement: Text S1 — Details regarding the selection of target tissue and the definition of physicochemical constraints. A supplementary text for Figure S4 is also included. (DOCX) [file pcbi.1002769.s007.docx]

# Functional Analysis of Metabolic Channeling and Regulation in Lignin Biosynthesis: A Computational Approach

### Yun Lee^1,3^, Luis Escamilla-Treviño^3,4^, Richard A. Dixon^3,4^, Eberhard O. Voit ^2,3§^

^1^The Interdisciplinary Bioengineering Program and ^2^The Wallace H. Coulter Department of Biomedical Engineering, Georgia Institute of Technology and Emory University, 313 Ferst Drive, Atlanta, Georgia 30332, USA;

^3^BioEnergy Sciences Center (BESC), Oak Ridge, Tennessee 37831, USA;

^4^Plant Biology Division, Samuel Roberts Noble Foundation, Ardmore, Oklahoma 73401, USA

^§^Corresponding author

**Text S1**

**Selection of target tissue in a wild-type *Medicago* species**

The parameter values for each model instantiation were selected in such a way that the nominal steady state is representative of wild-type *Medicago*. In this study, we chose alfalfa (*Medicago sativa* L.) as the model organism because of its extensive depository of perturbation-response data, including the results of experiments in which seven lignin biosynthetic enzymes were genetically down-regulated and the lignin content and composition in several stem internodes of each down-regulated line were determined [[1](#_ENREF_1)]. Of note, this list of down-regulated genes does not include CCR1 and CCR2, which have only recently been analyzed with *Medicago truncatula* lines harboring transposon insertions in *CCR1* and *CCR2* [[2](#_ENREF_2)]. In order to minimize the discrepancy in biological context, we thus chose the sixth internode (numbered from top to bottom) of stem as the target tissue because this is where the lignin content and composition were determined for the *ccr1* and *ccr2* mutants [[2](#_ENREF_2)].

**Physicochemical constraints on steady-state fluxes**

Two pieces of information for this specific stem internode in a wild-type alfalfa plant can be exploited, along with other stoichiometric and thermodynamic constraints, to define a biologically realistic set of reaction rates (or fluxes) at the nominal steady state. First, wild-type alfalfa is known to contain principally S and G lignin, while the incorporation of 5-hydroxyconiferyl alcohol into lignin polymer only occurs in COMT-deficient plants [[3](#_ENREF_3),[4](#_ENREF_4)]. Thus, it is reasonable to assume that the target tissue in a wild-type alfalfa plant has evolved to maximize the production of G and S lignin at the expense of 5-hydroxyguaiacyl (5HG) lignin. Second, the S/G ratio, that is, the ratio of sinapyl (S) to guaiacyl (G) lignin monomers, is equal to 0.58 [[1](#_ENREF_1)]. As in our previous work [[5](#_ENREF_5)], this information can be translated into a “proportionality constraint” on the fluxes leading to G and S lignin. Combining this information, we can represent the set *P* of steady-state fluxes, defined as *m*-dimensional real vectors, in the following mathematical format

$$P=\{v|\mathbf{c}^{T}\mathbf{v}=f^{*},\text{ }\mathbf{b}^{T}\mathbf{v}=0,\text{ }\mathbf{Nv}=\mathbf{0},\text{ }v_{1}=1,\text{ }v_{i}\geq l_{i},\text{ }i=1,...,m\}.$$

The definition for each of the five conditions is listed below:

1. **c***^T^* **v** = *f**: This condition states that the sum of fluxes leading to G and S lignin (**c***^T^***v**) should be fixed at a value *f**, which is obtained by solving the following linear programming problem:

$$\begin{matrix} f^{*}=\max\mathbf{c}^{T}\mathbf{v} \\ \text{subject to }\mathbf{b}^{T}\mathbf{v}=0 \\ \mathbf{Nv}=0 \\ v_{1}=1 \\ v_{i}\geq l_{i} \\ i=1,...,m \end{matrix}$$

1. **b***^T^* **v** = 0: This equation defines the proportionality constraint on the fluxes leading to G and S lignin as described above. Elements in **b** are determined by the specific value of the S/G ratio.
2. **Nv** = **0**: This condition describes the conservation of mass, or mass balance. **N** is an *n*×*m* stoichiometric matrix for a given design with *n* dependent variables and *m* reactions.
3. *v*_1_ = 1: As no reaction in the model is known to be reversible, with the exception of HCT, setting the input flux (*v*_1_) to 1 ensures that all the fluxes are less than or equal to one. In other words, this condition works as a means of standardization.
4. *v_i_* ≥ *l_i_*: This condition defines the lower bounds on individual reactions. For the *i*-th flux *v_i_*, it is bounded from below by *l_i_*. Here, we assume that all the enzymatic reactions and transport processes are irreversible and thus have a lower bound of zero. The only exception is the process that represents the transport of 5-hydroxyconiferyl alcohol into the cell wall, for which we arbitrarily choose 0.01 as the lower bound to prevent its value from becoming too small when solving for *f**.

Remarks regarding Figure S4

For each configuration, the steady-state flux distribution was initially sampled from a solution space that characterizes the metabolic phenotype of a wild-type *Medicago* species (see above for a list of constraints used to define the solution space). However, we were not able to constrain the flow *within* the network because of the lack of intracellular flux measurements, thereby leading to high variation in the flux distribution across model instantiations. In this case, knocking out CCR1 would change the S/G ratio only when at least one of the fluxes leading to S lignin (*e.g.* *v*_7_ and *v*_8_ in Figure S4A) or G lignin (*e.g.* *v*_4_ in Figure S4A) is altered compared to its pre-perturbation or wild-type level. Consistent with the results shown in Figure 5 of the text, only the configurations A, B, E, F, I, and O have model instantiations that yield such simulation results. In order words, it is the topological configuration—not the flux distribution—that determines whether the S/G ratio would alter upon perturbation of CCR1.

In the simulation of a *ccr2* knockout mutant, different subsets of independent variables are changed depending on whether hierarchical regulation is effective or not. Specifically, as mentioned in the main text, hierarchical regulation has been suggested to be effective through an indirect activation of CCR1 and CCoAOMT in the *ccr2* knockout mutant. To test whether the consideration of this effect would lead to different predictions regarding the S/G ratio for *ccr2* knockout, we performed two simulations: (1) CCR2 down-regulation alone; and (2) CCR2 down-regulation plus CCR1 and CCoAOMT up-regulation. The model equations are the same in both simulations; and only different subsets of independent variables are altered.

References

1. Chen F, Reddy MSS, Temple S, Jackson L, Shadle G, et al. (2006) Multi-site genetic modulation of monolignol biosynthesis suggests new routes for formation of syringyl lignin and wall‐bound ferulic acid in alfalfa (*Medicago sativa* L.). Plant J 48: 113-124.

2. Zhou R, Jackson L, Shadle G, Nakashima J, Temple S, et al. (2010) Distinct cinnamoyl CoA reductases involved in parallel routes to lignin in *Medicago truncatula*. Proc Natl Acad Sci USA 107: 17803-17808.

3. Guo D, Chen F, Inoue K, Blount JW, Dixon RA (2001) Downregulation of caffeic acid 3-*O*-methyltransferase and caffeoyl CoA 3-*O*-methyltransferase in transgenic alfalfa: impacts on lignin structure and implications for the biosynthesis of G and S lignin. Plant Cell 13: 73-88.

4. Marita JM, Ralph J, Hatfield RD, Guo D, Chen F, et al. (2003) Structural and compositional modifications in lignin of transgenic alfalfa down-regulated in caffeic acid 3-*O*-methyltransferase and caffeoyl coenzyme A 3-*O*-methyltransferase. Phytochemistry 62: 53-65.

5. Lee Y, Chen F, Gallego-Giraldo L, Dixon RA, Voit EO (2011) Integrative analysis of transgenic alfalfa (*Medicago sativa* L.) suggests new metabolic control mechanisms for monolignol biosynthesis. PLoS Comput Biol 7: e1002047.
